# Microcurrent stimulation induces cell death in p53-mutant and 5-FU-resistant breast cancer

**DOI:** 10.1016/j.jbc.2025.110414

**Published:** 2025-06-24

**Authors:** Tomohito Tanihara, Yuya Yoshida, Takashi Ogino, Yuma Terada, Fumiaki Tsurusaki, Keika Hamasaki, Kaita Otsuki, Kohei Fukuoka, Kosuke Oyama, Akito Tsuruta, Kengo Hamamura, Kouta Mayanagi, Satoru Koyanagi, Yuichi Murakami, Mayumi Ono, Michihiko Kuwano, Shigehiro Ohdo, Naoya Matsunaga

**Affiliations:** 1 Faculty of Pharmaceutical Sciences, Department of Clinical Pharmacokinetics, Kyushu University, Fukuoka, Japan; 2 Faculty of Pharmaceutical Sciences, Department of Pharmaceutics, Kyushu University, Fukuoka, Japan; 3 Faculty of Advanced Engineering, Department of Biological Science and Technology, Tokyo University of Science, Shinjuku-ku, Tokyo, Japan; 4 Faculty of Pharmaceutical Sciences, Department of Drug Discovery Structural Biology, Kyushu University, Fukuoka, Japan; 5 Basic Medical Research Unit, St. Mary's Research Center, Kurume, Fukuoka, Japan

**Keywords:** 5-fluorouracil, chemoresistance, MDA-MB-231 cells, p53, microcurrent, breast cancer, cell death, flow cytometry, apoptosis, reactive oxygen species (ROS)

## Abstract

5-Fluorouracil (5-FU) is a commonly used chemotherapeutic agent for breast cancer. Its efficacy relies on the function of p53, and mutations in p53 contribute to the development of resistance during 5-FU chemotherapy. Here, we report that microcurrent stimulation (MCS) of a p53-mutant breast cancer cell line induces p53-mediated cell death. Although MDA-MB-231 and MDA-MB-468 cells, both human breast cancer cell lines, are less sensitive to 5-FU due to p53 mutations, MCS (300 **μ**A for 30 min) induced apoptosis in these cells and improved the antitumor effect of 5-FU in tumor-bearing mice. MCS-induced apoptosis was mediated by an increase in intracellular Cu^2+^ ions and reactive oxygen species, along with the concurrent transcriptional enhancement of pro-apoptotic genes by p53. Furthermore, MCS induced apoptosis in MDA-MB-231 cells that had developed resistance to 5-FU and inhibited tumor growth in tumor-bearing mice with reduced 5-FU sensitivity. These findings suggest that an approach involving MCS could serve as a foundation for developing breast cancer treatment strategies to overcome p53 mutations.

Breast cancer is the most common carcinoma affecting women. Advances in diagnostics and treatment approaches have substantially increased the 5-year survival rate for patients, contributing to a favorable prognosis. Chemotherapy remains a major treatment option, particularly for first-episode and many recurrent breast cancers, and is employed in multiple-drug combinations, including anthracyclines, taxanes, and fluorouracil (FU) (1, 2). 5-FU and its prodrugs are commonly used in breast cancer chemotherapy; they exert their antitumor effects through various mechanisms, including the inhibition of thymidylate synthase and the misincorporation of 5-FU metabolites into RNA and DNA (3). However, the development of drug resistance during chemotherapy is often observed, which limits the clinical applicability of 5-FU.

The tumor suppressor gene p53 is a transcription factor that regulates critical biological processes. Known as the “guardian of the genome,” p53 responds to stresses such as DNA damage and inhibits tumorigenesis by inducing cell death, including apoptosis (4). Therefore, p53 plays a central role in the efficacy of many anticancer drugs, with 5-FU being highly dependent on p53 for its cytotoxic activity (5, 6, 7). However, mutations in the p53 gene have been observed in various cancer types, with missense mutations found in approximately 70% of cases with breast cancer (8). The most common p53 mutations are missense mutations, primarily occurring in the DNA-binding or tetramer domains of the p53 gene, which leads to a decrease in p53 transcriptional activity (9). These mutations prevent the induction of cell death in cancer cells in response to anticancer drugs (10). Additionally, p53 mutants are known to possess gain-of-function (GOF) activity, contributing to the proliferative and metastatic potential and stemness of cancer cells by interacting with various factors (11, 12, 13). Therefore, the induction of p53 by anticancer agents such as 5-FU presents a double-edged sword in chemotherapy for breast cancer, highlighting the need for therapies that can overcome p53 mutations.

Cancer is rarely treated *via* systemic therapy with anticancer agents alone. In most cases, local therapy is combined with anticancer agents for optimal treatment outcomes. While radiation has long been used as a form of local therapy, an alternating electric field device called OPTUNE was recently approved by the FDA, representing a potential alternative. OPTUNE treatment is employed for the treatment of glioblastoma, enhancing the effects of chemotherapy by providing electric field stimulation (200 kHz) for several hours a day in parallel with temozolomide administration (14). However, this treatment strategy puts a significant burden on the patient's quality of life, in addition to adverse effects on the brain. Electrical stimulation that does not exceed the *in vivo* potential change (∼500 μA) caused by neurotransmission is called microcurrent stimulation (MCS), which exerts minimal effects on membrane potential and generation of thermal energy. MCS is therefore a non-invasive, pain-free, and safe stimulation condition. Moreover, MCS can produce effects such as pain relief and wound healing after several minutes of stimulation (15, 16), leading to its use in sports medicine with a proven track record in human use. However, the molecular mechanisms through which MCS influences cells remain elusive, and its effects on tumors are unknown.

The purpose of this study was to understand the effect of 5-FU, an anticancer drug that strongly induces p53, on the properties of breast cancer cells with p53 mutations and to establish an effective therapeutic strategy. Human breast cancers with mutations in p53 showed a poor response to 5-FU; in particular, in MDA-MB-231 cells, the administration of 5-FU contributed to tumor growth. However, treating MDA-MB-231 cells with MCS under previously identified conditions (17) led to apoptosis through the transcriptional activation of p53. Additionally, the transcriptional activation of mutant p53 by MCS sensitized the cells to 5-FU, enhancing its ability to induce apoptosis. This combined effect resulted in a marked antitumor response, inhibiting 5-FU-derived tumor growth. Our findings highlight the potential risks of existing 5-FU-containing breast cancer regimens and underscore the potential of combining MCS with chemotherapy as a therapeutic strategy for breast cancer.

## Results

### MCS enhances antitumor effects of 5-FU in MDA-MB-231 cells

**Figure 1:**
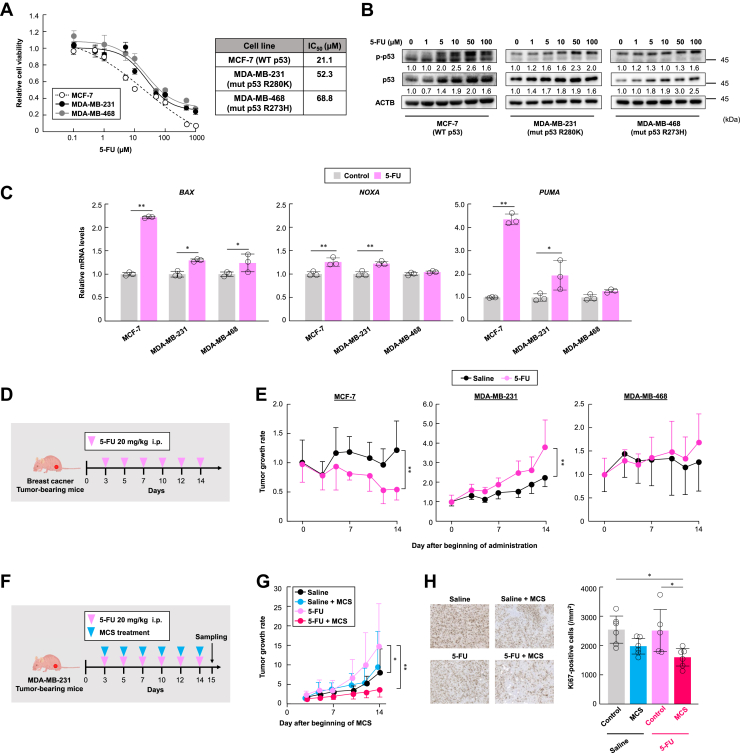
**MCS improves resistance to 5-FU in cells and enhances antitumor effects in MDA-MB-231 cells.***A*, cell viability of MCF-7, MDA-MB-231, and MDA-MB-468 cells exposed to 5-FU. Cells were treated with 5-FU at concentrations ranging from 0 to 1000 μM for 72 h. The *right panel* shows the IC_50_ values for each cell line, with the 5-FU untreated group set at 1.0. Values are presented as the mean ± S.D. (n = 4). *B*, protein levels of p53 and phosphorylated p53 (p-p53) after treatment with 5-FU at concentrations ranging from 0 to 100 μM for 48 h in MCF-7, MDA-MB-231, and MDA-MB-468 cells. The value of the 5-FU untreated group is set at 1.0. The uncropped image of p53 and p-p53 proteins in the cells is illustrated in Figure S13*A*. *C*, mRNA levels of p53 target pro-apoptotic genes *BAX, NOXA,* and *PUMA* after treatment with 5-FU at a concentration of 10 μM for 48 h in MCF-7, MDA-MB-231, and MDA-MB-468 cells. The value of the 5-FU untreated group is set at 1.0. Values are presented as the mean ± S.D. (n = 3). ∗*p* < 0.05, ∗∗*p* < 0.01 (ANOVA with a Tukey–Kramer *post hoc* test). *D*, schematic experimental procedure for treating breast cancer transplanted mice with 5-FU alone for 2 weeks 5-FU (20 mg/kg/mouse) was administered intraperitoneally to tumor-bearing mice three times a week. The image of the nude mouse is from TogoTV (©2016 DBCLS TogoTV, CC-BY-4.0 https://creativecommons.org/licenses/by/4.0/deed.ja). *E*, influence of 5-FU treatment on tumor growth rate in MCF-7, MDA-MB-231, and MDA-MB-468-transplanted mice. All breast cancer cells were subcutaneously inoculated into the fat pads of mice. Tumor volume of day 0 is set at 1.0. Values are presented as the mean ± S.D. (n = 6–8). ∗∗*p* < 0.01 indicates significant differences between the groups (*F*_11,78_ = 13.527, *p* < 0.001 for 4T1; *F*_11,72_ = 3.833, *p* < 0.001 for MCF-7; *F*_11,66_ = 11.73, *p* < 0.001; ANOVA with a Tukey–Kramer *post hoc* test). *F*, schematic of the experimental procedure for treating MDA-MB-231 transplanted mice with a combination of MCS and 5-FU for 2 weeks 5-FU (20 mg/kg/mouse) was administered intraperitoneally to tumor-bearing mice three times a week. MCS treatment was performed after 5-FU administration. *G*, influence of combined MCS and 5-FU treatment on tumor growth rate in MDA-MB-231-transplanted mice. (Saline: n = 8, Saline + MCS: n = 6, 5-FU: n = 8, 5-FU + MCS: n = 8). MDA-MB-231 cells were subcutaneously inoculated into the fat pads of mice. Tumor volume of day 0 is set at 1.0. Values are presented as the mean ± S.D. (n = 6–8). ∗∗*p* < 0.01 and ∗*p* < 0.05 indicate significant differences between the groups (*F*_*23,156*_ = 4.514, *p <* 0.001; ANOVA with a Tukey–Kramer *post hoc* test). *H*, immunohistochemical staining of Ki-67 in MDA-MB-231 tumors. Ki-67 signals were visualized using 3,3′-diaminobenzidine (*brown*), and nuclei were stained with hematoxylin (*blue*). Values are presented as the mean ± S.D. (n = 5, 6). ∗*p* < 0.05 indicates a significant difference between the groups (*F*_*3,19*_ = 4.735, *p =* 0.012; ANOVA with a Tukey–Kramer *post hoc* test).

5-FU depends on p53 to exert its cytotoxic effects (4, 5, 6). We first measured the IC_50_ and p53 transcriptional function in response to 5-FU exposure in three human breast cancer cell lines with different p53 statuses: MCF-7 (WT-p53), MDA-MB-231 (R280K mut-p53), and MDA-MB-468 (R273H mut-p53) (18). MDA-MB-231 and MDA-MB-468 exhibited higher IC_50_ values than MCF-7, which expresses wild-type p53 (Fig. 1*A*). We also evaluated the protein levels of p53 and p53 phosphorylated at Ser15, the initiator of stabilization and transcriptional activation (19, 20) upon 5-FU exposure in these cells.

The increase in p53 and phosphorylated p53 at Ser15 (p-p53) was strongly induced in MCF-7 cells, even at concentrations of 5 to 10 μM of 5-FU, while no comparable increase was observed in MDA-MB-231 and MDA-MB-468 cells (Fig. 1*B*). Moreover, the mRNA expression of *BAX* and *PUMA*, except for *NOXA*, two pro-apoptotic genes transcriptionally regulated by p53, was significantly increased by exposure to 10 μM 5-FU in MCF-7 cells (Fig. 1*C*). These results suggest that the activation and stabilization of p53 through phosphorylation, sufficient to induce cell death, are crucial for 5-FU to exert its cytotoxic effect in breast cancer cells.

We next evaluated the sensitivity of these breast cancer cells to 5-FU *in vivo*. Treatment of mice with 5-FU (20 mg/kg) resulted in tumor shrinkage only in MCF-7 tumors, whereas in MDA-MB-231 tumors, tumor growth was observed inversely (Fig. 1, *D* and *E*). Therefore, we evaluated the combined effect of 5-FU (20 mg/kg) and MCS (300 μA, 30 min) on tumors transplanted with MDA-MB-231 cells (Fig. 1*F*). While 5-FU alone had no antitumor effect, the combination of 5-FU and MCS exhibited a strong antitumor effect (Fig. 1*G*). Additionally, Ki-67 staining, an indicator of cancer cell proliferation, was lowest in tumors treated with 5-FU and MCS (Fig. 1*H*). Thus, although 5-FU exhibited poor efficacy against breast cancer cells with p53 mutations, the combination of 5-FU and MCS has a strong antitumor effect against these cells.

### MCS induces mutant p53 activation by stimulating the influx of metal ions

**Figure 2:**
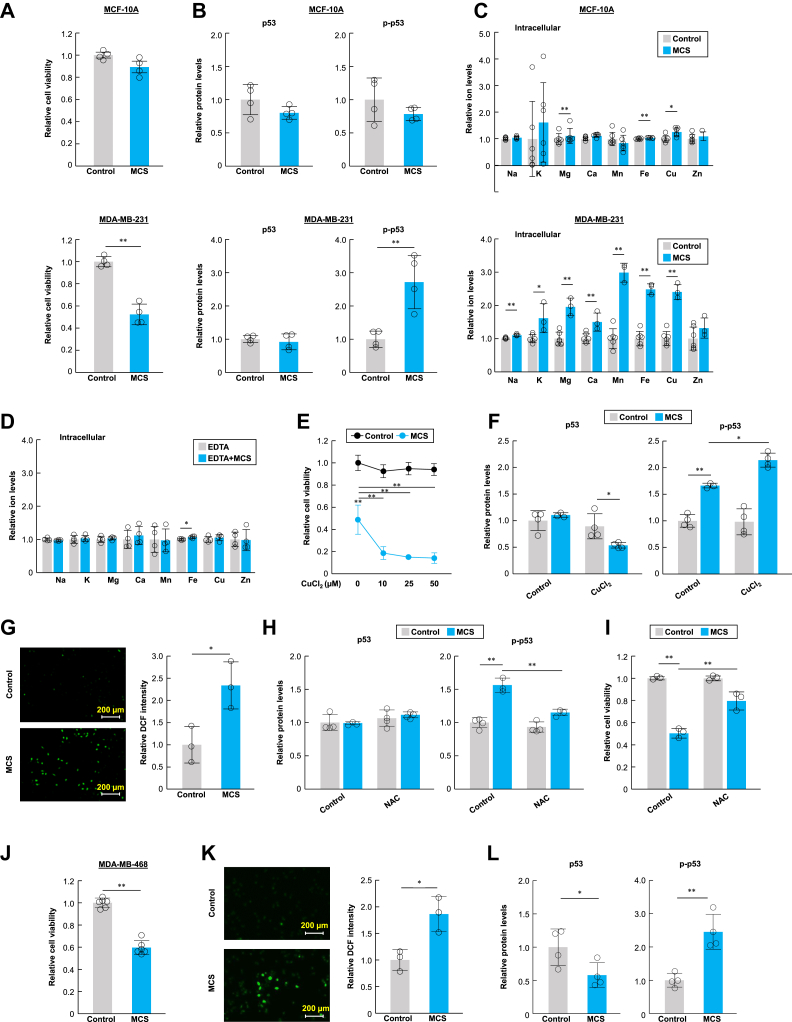
**MCS induces apoptosis *via* activation of mutant p53 in MDA-MB-231 cells.***A*, effect of MCS treatment on MCF-10A and MDA-MB-231 cell viability. Cell viability was assessed 48 h after MCS treatment. The value of the non-MCS-treated group was set at 1.0. Values are presented as the mean ± S.D. (n = 4). ∗∗*p* < 0.01 indicates significant differences between two groups (*t*_*6*_ = 9.157 for MDA-MB-231; *t*_*6*_ = 10.092 for MDA-MB-468; Student’s *t* test). *B*, the protein levels of p53 and p-p53 in MCF-10A and MDA-MB-231 cells. The value of the non-MCS-treated group is set at 1.0. Values are presented as the mean ± S.D. (n = 4). ∗∗*p* < 0.01 indicates a significant difference between the two groups (*t*_*6*_ = 4.098 for p-p53; Student’s *t* test). *C*, effect of MCS treatment on the intracellular concentrations of metal ions in MCF-10A and MDA-MB-231 cells. The value of non-MCS-treated group is set at 1.0. Values are presented as the mean ± S.D. (n = 3–6). ∗*p* < 0.05 and ∗∗*p* < 0.01 indicate significant differences from each control (MCF-10A: Intracellular; *t*_*10*_ = 3.323 for Ca; *t*_*10*_ = 4.374 for Fe; *t*_*10*_ = 3.192 for Cu. MDA-MB-231: Intracellular; *t*_*7*_ = 6.157 for Na; *t*_*7*_ = 3.429 for K; *t*_*7*_ = 6.250 for Mg; *t*_*7*_ = 3.726 for Ca; *t*_*7*_ = 9.594 for Mn; *t*_*7*_ = 10.317 for Fe; *t*_*7*_ = 9.197 for Cu; Student’s *t* test). *D*, influence of the metal chelator EDTA (1 mM) on the MCS-induced upregulation of intracellular metal ions in MDA-MB-231 cells. The value of non-MCS-treated group is set at 1.0.Values are presented as the mean ± S.D. (n = 4). ∗*p* < 0.05 indicates a significant difference between the two groups (*t*_*6*_ = 3.623 for Fe; Student’s *t* test). *E*, influence of Cu^2+^ addition to the culture medium on the inhibition of MCS-induced cell proliferation in MDA-MB-231 cells. Cell viability was assessed 48 h after MCS treatment in the presence of CuCl_2_, with the non-MCS-treated group set at a value of 1.0. Values are presented as the mean ± S.D. (n = 3). ∗∗*p* < 0.01 indicates significant differences between the two groups (*F*_*4,10*_ = 73.535, *p* < 0.001; ANOVA with a Tukey–Kramer *post hoc* test). *F*, influence of Cu^2+^ (25 μM) addition to the culture medium on the protein levels of p-p53 in MDA-MB-231 cells. The non-MCS-treated without Cu^2+^ addition group is set at a value of 1.0. Values are presented as the mean ± S.D. (n = 3–4). ∗∗*p* < 0.01 indicates a significant difference between the groups (*F*_*3,12*_ = 23.885, *p* < 0.001 for p-p53; ANOVA with a Tukey–Kramer *post hoc* test). *G*, the intracellular ROS levels in MDA-MB-231 cells at 30 min after MCS. The value of the non-MCS-treated group is set at 1.0. Values are presented as the mean ± S.D. (n = 3). ∗*p* < 0.05 indicates a significant difference between the two groups (*t*_*4*_ = 3.449; Student’s *t* test). *H*, influence of N-acetylcysteine (NAC; 5 mM) on the protein levels of p53 and p-p53 in MDA-MB-231 cells. The non-MCS-treated without NAC is set to a value of 1.0. Values are presented as the mean ± S.D. (n = 3–4). ∗∗*p* < 0.01 indicates a significant difference between the groups (*F*_*3,10*_ = 41.657, *p* < 0.001 for p-p53; ANOVA with a Tukey–Kramer *post hoc* test). *I*, influence of NAC (5 mM) on MCS-induced inhibition of cell proliferation. Cell viability was assessed 48 h after MCS treatment in the presence of NAC. The non-MCS-treated without NAC is set to a value of 1.0. Values are presented as the mean ± S.D. (n = 3). ∗∗*p* < 0.01 indicates a significant difference between the two groups (*F*_*3,8*_ = 72.645, *p* < 0.001; ANOVA with a Tukey–Kramer *post hoc* test). *J*, effect of MCS treatment on MDA-MB-468 cell viability. Cell viability was assessed 48 h after MCS treatment. The value of the non-MCS-treated group was set at 1.0. Values are presented as the mean ± S.D. (n = 6). ∗∗*p* < 0.01 indicates significant differences between the two groups (*t*_*6*_ = 10.092; Student’s *t* test). *K*, the intracellular ROS levels in MDA-MB-468 cells at 30 min after MCS. The value of the non-MCS-treated group is set at 1.0. Values are presented as the mean ± S.D. (n = 3). ∗*p* < 0.05 indicates a significant difference between the two groups (*t*_*4*_ = 3.904; Student’s *t* test). *L*, the protein levels of p53 and p-p53 in MDA-MB-468 cells. The value of the non-MCS-treated group is set at 1.0. Values are presented as the mean ± S.D. (n = 4). ∗∗*p* < 0.01 and ∗*p* < 0.05 indicate significant differences between the two groups (*t*_*6*_ = 2.548 for p53; *t*_*6*_ = 5.161 for p-p53; Student’s *t* test).

Since MCS enhanced the antitumor effect of 5-FU on MDA-MB-231, we hypothesized that MCS affected the p53 pathway, a target of the cell-killing effect of 5-FU. To confirm this hypothesis, we first evaluated cell viability and p53 and p-p53 expression following MCS treatment in MDA-MB-231 cells and the non-neoplastic mammary cell line, MCF-10A. Reduced cell viability and increased p-p53 protein were observed only in MCS-treated MDA-MB-231 cells (Fig. 2, *A* and *B*). Weak current stimulation (μA level) induces various biological responses *via* the intracellular influx of metal ions (21, 22). Inductively coupled plasma mass spectrometry (ICP-MS) was performed to measure changes in the balance of metal ions inside MCF-10A and MDA-MB-231 cells. MCS slightly increased the intracellular concentrations of calcium (Ca), ferric (Fe), and copper (Cu) ions in MCF-10A cells (Fig. 2*C*). However, in MDA-MB-231 cells, MCS increased various intracellular metal ions, especially divalent metal ions such as manganese (Mn), Fe, and Cu (Fig. 2*C*). Therefore, we evaluated the effect of MCS by adding ethylenediaminetetraacetic acid (EDTA), a chelating agent for divalent metal ions, to the culture medium. EDTA suppressed the increase in intracellular metal ions induced by MCS in MDA-MB-231 cells (Fig. 2*D*). Additionally, Ca^2+^, Mg^2+^, Mn^2+^, Fe^2+^, and Cu^2+^ were added to the culture medium, and cell survival rates after MCS treatment were evaluated (Fig. S1*A*). These results suggest that the cell proliferation inhibitory effect of MCS was enhanced only in cells treated with Cu^2+^ ions (Fig. 2*E*). The addition of Cu^2+^ ions also increased the levels of p-p53 protein induced by MCS (Fig. 2*F*). Exposure to EDTA, a chelator of divalent metal ions, or tetra thiomolybdate (TTM), a specific chelator of Cu^2+^ ions, consistently abolished the MCS-induced increase in p-p53 and the inhibitory effect on cell proliferation (Figs. S2, *A* and *B* and S3, *A* and *B*). Furthermore, the effect of MCS was eliminated when SLC11A2, a transporter that directly takes up Cu^2+^ ions (23), was knocked out (Fig. S4*A*). These results suggest that MCS induces p53 phosphorylation *via* the influx of Cu^2+^ ions.

Excessive Cu^2+^ ion influx into cells induces reactive oxygen species (ROS) accumulation *via* the Fenton reaction, which triggers DNA damage and subsequent p53-dependent apoptosis (24, 25, 26, 27, 28). We measured ROS production in MDA-MB-231 cells treated with MCS and observed increased intracellular ROS (Fig. 2*G*). Furthermore, N-acetylcysteine, a ROS scavenger, attenuated the increase in p-p53 and inhibited cell proliferation without causing Cu^2+^ ion influx into the cells (Figs. 2, *H* and *I*, and S5). These results suggest that MCS-induced ROS accumulation and the subsequent p53-dependent apoptosis are downstream effects of Cu^2+^ ion influx.

MCS also exhibited an inhibitory effect on cell proliferation in MDA-MB-468 cells (Fig. 2*J*), leading to ROS accumulation and an increase in p-p53 levels in MDA-MB-468 cells (Fig. 2, *K* and *L*). These results suggest that the cell death induction mechanism mediated by MCS is similar in both MDA-MB-231 and MDA-MB-468 cells.

### MCS induces apoptosis *via* p53 transcriptional activation

**Figure 3:**
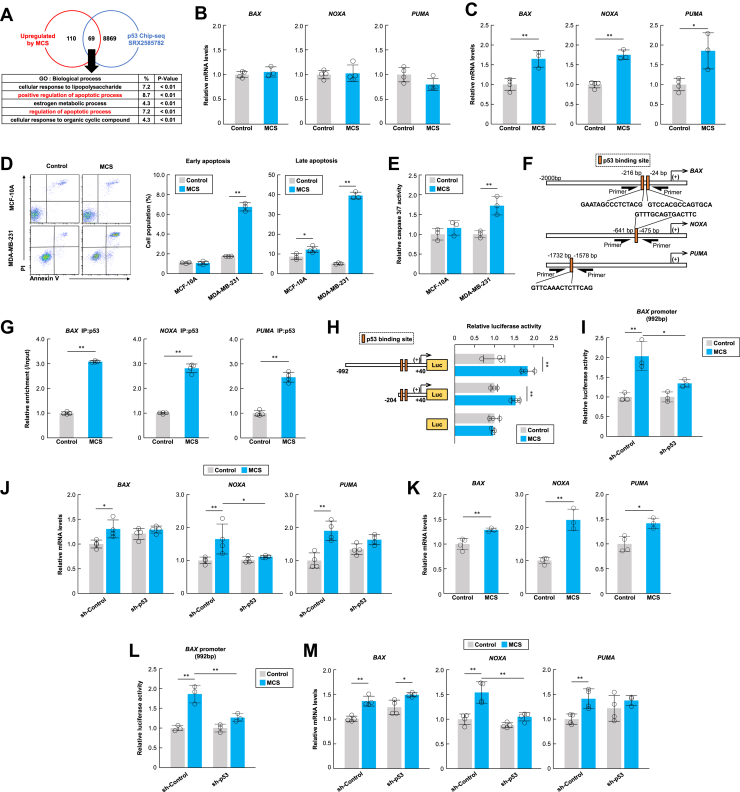
**MCS enhances apoptosis induction by 5-FU in MDA-MB-231 cells.***A*, functional analysis of genes overlapping in two datasets: (1) differential expression between non-MCS-treated and MCS-treated MDA-MB-231 cells, and (2) ChIP-seq data (Antigen: p53, Cell line: MDA-MB-231, criteria: Binding score ≥50) using the gene Ontology Resource in the DAVID system. *B*, *C*, mRNA levels of p53 target pro-apoptotic genes *BAX, NOXA,* and *PUMA* in MCF-10A and MDA-MB-231 cells at 24 h after MCS. The value of the non-MCS-treated group is set at 1.0. Values are presented as the mean ± S.D. (n = 3–4). ∗*p* < 0.05 and ∗∗*p* < 0.01 indicate significant differences between the two groups (*t*_*5*_ = 4.825 for *BAX*; *t*_*5*_ = 9.861 for *NOXA*; *t*_*5*_ = 3.554 for *PUMA*; Student’s *t* test). *D*, flow cytometry analysis of annexin V in MCF-10A and MDA-MB-231 cells at 24 h after MCS. The right panel shows the difference in the ratio of Annexin-FITC^+^/PI^-^ (early apoptosis) to Annexin-FITC^+^/PI^+^ (late apoptosis) cell populations between non-MCS-treated and MCS-treated breast cell lines. Values are presented as the mean ± S.D. (n = 3–4). ∗*p* < 0.05 and ∗∗*p* < 0.01 indicate significant differences from each control group (*t*_*5*_ = 3.036 for late apoptosis in MCF-10A; *t*_*4*_ = 20.776 for early apoptosis in MDA-MB-231; *t*_*4*_ = 34.180 for late apoptosis in MDA-MB-231; Student’s *t* test). *E*, caspase-3/7 activity in MDA-MB-231 cells at 24 h after MCS. Values are presented as the mean ± S.D. (n = 3). The value of the non-MCS-treated group is set at 1.0. ∗∗*p* < 0.01 indicates a significant difference between the two groups (*t*_*4*_ = 5.728; Student’s *t* test). *F*, schematic representation of the human *BAX, NOXA,* and *PUMA* genes. The numbers indicate the distance from the transcription start site (+1). Black rectangles represent p53 binding sites. *G*, ChIP analysis of MCS-induced changes in the binding amount of p53 protein to the upstream regions of *BAX, NOXA,* and *PUMA* genes in MDA-MB-231 cells. The value of the non-MCS-treated group is set at 1.0. Values are presented as the mean ± S.D. (n = 4). ∗∗*p* < 0.01 indicates significant differences between the two groups (*t*_*6*_ = 71.814 for *BAX*; *t*_*6*_ = 19.936 for *NOXA*; *t*_*6*_ = 12.871 for *PUMA*; Student’s *t* test). *H*, the promoter activities of the *BAX* luciferase reporter in MDA-MB-231 cells at 24 h after MCS. The value of the non-MCS-treated group is set at 1.0.Values are presented as the mean ± S.D. (n = 3). ∗∗*p* < 0.01 indicates a significant difference between the two groups (*F*_5,12_ = 17.8, *p* < 0.001; ANOVA with a Tukey–Kramer *post hoc* test). *I*, effect of *p53* knockdown on MCS-induced upregulation of *BAX* luciferase reporter activity in MDA-MB-231 cells. The expression of the p53 protein in the cells is illustrated in Figure S13*B*. The value of the non-MCS-treated group is set at 1.0. Values are presented as the mean ± S.D. (n = 3). ∗*p* < 0.05 and ∗∗*p* < 0.01 indicate significant differences between the groups (*F*_3,8_ = 16.7, *p* = 0.0008; ANOVA with a Tukey–Kramer *post hoc* test). *J*, effect of *p53* knockdown on MCS-induced pro-apoptotic gene transcription in MDA-MB-231 cells. The value of the sh-Control non-MCS-treated group is set at 1.0. Values are presented as the mean ± S.D. (n = 4). ∗*p* < 0.05 and ∗∗*p* < 0.01 indicate significant differences between the groups (*F*_3,12_ = 5.595, *p* = 0.0123 for *BAX*; *F*_3,12_ = 12.731, *p* = 0.0005 for *NOXA*; *F*_3,12_ = 6.549, *p* = 0.0072 for *PUMA*; ANOVA with a Tukey–Kramer *post hoc* test). *K*, mRNA levels of p53 target pro-apoptotic genes *BAX, NOXA,* and *PUMA* in MDA-MB-468 cells at 24 h after MCS. The value of the non-MCS-treated group is set at 1.0. Values are presented as the mean ± S.D. (n = 3–4). ∗*p* < 0.05 and ∗∗*p* < 0.01 indicate significant differences between the two groups (*t*_*5*_ = 4.074 for *BAX*; *t*_*5*_ = 7.383 for *NOXA*; *t*_*5*_ = 4.021 for *PUMA*; Student’s *t* test). *L*, effect of *p53* knockdown on MCS-induced upregulation of *BAX* luciferase reporter activity in MDA-MB-468 cells. The expression of the p53 protein in the cells is illustrated in Figure S13*C*. The value of the non-MCS-treated group is set at 1.0. Values are presented as the mean ± S.D. (n = 3).∗∗*p* < 0.01 indicates significant differences between the groups (*F*_3,8_ = 25.975, *p* = 0.0002; ANOVA with a Tukey–Kramer *post hoc* test). *M*, effect of *p53* knockdown on MCS-induced pro-apoptotic gene transcription in MDA-MB-468 cells. The value of the sh-Control non-MCS-treated group is set at 1.0. Values are presented as the mean ± S.D. (n = 3–4). ∗*p* < 0.05 and ∗∗*p* < 0.01 indicate significant differences between the groups (*F*_3,12_ = 19.236, *p* < 0.001 for *BAX*; *F*_3,12_ = 20.162, *p* < 0.001 for *NOXA*; *F*_3,11_ = 3.941, *p* = 0.0392 for *PUMA*; ANOVA with a Tukey–Kramer *post hoc* test).

p53 regulates the expression of various genes; however, the missense mutation of p53 in MDA-MB-231 cells reduces p53 binding to DNA, suppressing the transcription of target genes (29). We performed RNA-seq on MDA-MB-231 cells treated with MCS to evaluate the biological processes contributing to the transcription of genes promoted by p53 and found that MCS may induce apoptosis *via* p53 in MDA-MB-231 cells (Fig. 3*A*). Next, we measured the expression of *BAX, NOXA*, and *PUMA* after MCS treatment in MCF-10A and MDA-MB-231 cells. All pro-apoptotic genes were increased following MCS treatment only in MDA-MB-231 cells (Fig. 3, *B* and *C*). Additionally, we evaluated the effect of MCS on cell apoptosis in MCF-10A and MDA-MB-231 cells and found that MCS increased caspase activity and the number of apoptotic cells in MDA-MB-231 cells (Fig. 3, *D* and *E*). We performed chromatin immunoprecipitation (ChIP) on MCS-treated MDA-MB-231 cells to determine the amount of p53 binding to the promoter regions of *BAX, NOXA*, and *PUMA* (Fig. 3*F*) and observed an increase in p53 binding to p53 binding sequences on the promoter of all three genes following MCS treatment (Fig. 3*G*). In addition, luciferase reporter vectors containing the *BAX* promoter region were created, and the luciferase activity following MCS treatment was assessed in MDA-MB-231 cells. The results suggest a p53-binding sequence-dependent increase in luciferase activity by MCS (Fig. 3*H*), and the effect of MCS was attenuated by the knockdown of the p53 gene (Fig. 3*I*).

Furthermore, the transcriptional activation of pro-apoptotic genes observed with MCS was inhibited by p53 knockdown and EDTA exposure in MDA-MB-231 cells (Figs. 3*J*, S2*C*). In MDA-MB-468 cells, MCS induced the transcriptional activation of pro-apoptotic genes (Fig. 3*K*), but these effects were suppressed by p53 knockdown (Fig. 3, *L* and *M*). These results suggest that MCS induces apoptosis in MDA-MB-231 and MDA-MB-468 cells through enhanced transcriptional activity of p53 and the transcription of pro-apoptotic genes.

### MCS assists in apoptosis induction by 5-FU in MDA-MB-231 cells

**Figure 4:**
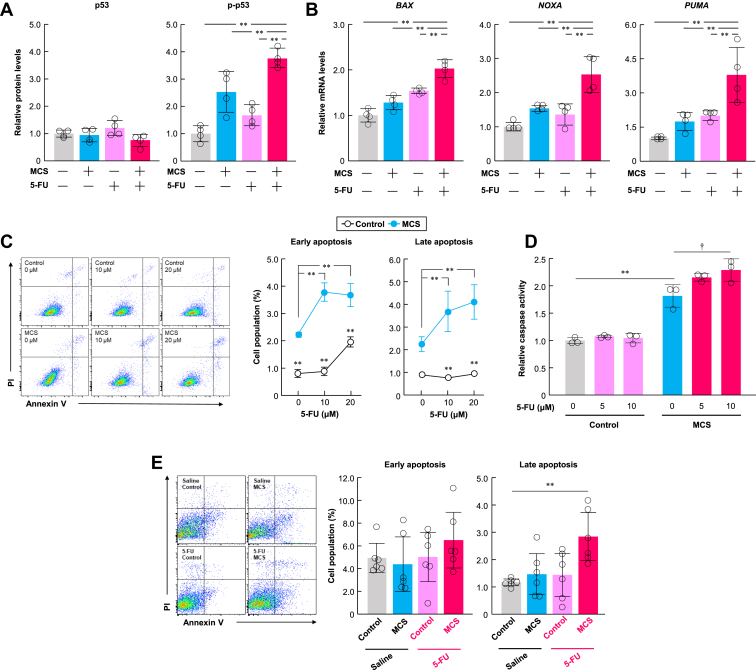
**MCS promotes metal ion influx and activates p53 in MDA-MB-231 cells.***A*, protein levels of p53, phosphorylated p53 (p-p53), and relative p53 phosphorylation levels in MDA-MB-231 cells treated with MCS and 5-FU (10 μM). The value of the non-MCS-treated without 5-FU treatment group is set at 1.0. Values are presented as the mean ± S.D. (n = 4). ∗∗*p* < 0.01 indicates a significant difference between the groups (*F*_*3,12*_ = 25.011, *p* < 0.001 for p-p53; *F*_*3,12*_ = 20.812, *p* < 0.001 for p-p53/p53; ANOVA with a Tukey–Kramer *post hoc* test). *B*, mRNA levels of p53 target genes *BAX, NOXA,* and *PUMA* in MDA-MB-231 cells treated with MCS and 5-FU (10 μM). The value of the non-MCS-treated without 5-FU treatment group is set at 1.0. Values are presented as the mean ± S.D. (n = 4). ∗∗*p* < 0.01 indicates a significant difference between the groups (*F*_*3,12*_ = 34.207, *p* < 0.001 for *BAX*; *F*_*3,12*_ = 17.404, *p* < 0.001 for *NOXA*; *F*_*3,12*_ = 13.322 for *PUMA*; ANOVA with a Tukey–Kramer *post hoc* test). *C*, influence of MCS on 5-FU-induced apoptosis in MDA-MB-231 cells. *Top panels* show the flow cytometry analysis of annexin V in non-MCS-treated or MCS-treated MDA-MB-231 cells in the presence or absence of 5-FU. The *panel* shows the difference in the ratio of FITC^+^/PI^-^ (early apoptosis) to FITC^+^/PI^+^ (late apoptosis) cell populations between non-MCS-treated or MCS-treated MDA-MB-231 cells in the presence or absence of 5-FU. Values are presented as the mean ± S.D. (n = 3). ∗*p* < 0.05 and ∗∗*p* < 0.01 indicate a significant difference between the groups (*F*_*5,12*_ = 79.575, *p* < 0.001 for early apoptosis; *F*_*5,12*_ = 27.343, *p* < 0.001 for late apoptosis; two-way ANOVA with the Tukey–Kramer *post hoc* test). *D,* caspase activity of MDA-MB-231 cells treated with 5-FU and MCS. The value of the non-MCS-treated without 5-FU treatment group is set at 1.0. Values are presented as the mean ± S.D. (n = 3).∗∗*p* < 0.01 indicates a significant difference between the groups (*F*_*5,12*_ = 62.821, *p* < 0.001; ANOVA with the Tukey–Kramer *post hoc* test). ^†^*p* < 0.05 indicates a significant difference from the MCS-treated 5-FU 0 μM group (*F*_*2,6*_ = 5.952, *p* < 0.0376; ANOVA with Dunnett’s *post hoc* test). *E*, influence of MCS on 5-FU-induced apoptosis in MDA-MB-231 tumors. The *right panel* shows the difference in the ratio of Annexin-FITC^+^/PI^-^ (early apoptosis) to Annexin-FITC^+^/PI^+^ (late apoptosis) cell populations in MDA-MB-231 tumors from each group. Values are presented as the mean ± S.D. (n = 6). ∗*p* < 0.05 indicates a significant difference from the saline group (*F*_*3,20*_ = 4.590, *p* = 0.0133; ANOVA with Dunnett’s *post hoc* test).

MCS induces apoptosis through p53 activation; therefore, we investigated the combined effects of 5-FU and MCS on p53-mediated apoptosis in MDA-MB-231 cells. MCS treatment increased p53 phosphorylation in these cells, peaking at 16 h post-treatment before decreasing over time (Fig. S6*A*). Consequently, we initially chose to sequence the treatments with 5-FU pretreatment followed by MCS treatment. The levels of p-p53 and the expression of p53 target genes were upregulated following additional MCS treatment after exposure to 5-FU (Fig. 4, *A* and *B*). A single exposure to 10 or 20 μM 5-FU had little effect on apoptosis in MDA-MB-231 cells (Fig. 4*C*). In contrast, additional MCS treatment after 5-FU exposure significantly increased the number of early and late apoptotic cells (Fig. 4*C*). Moreover, caspase activity induced by 5-FU was enhanced with subsequent MCS treatment (Fig. 4*D*). We also examined the induction of p53-mediated apoptosis when 5-FU exposure followed MCS treatment. The protein levels of p-p53 and caspase activity were the highest when 5-FU exposure occurred immediately after MCS (Fig. S6, *B* and *C*), resulting in significant inhibition of cell proliferation (Fig. S6*D*).

Based on these results, we evaluated the induction of apoptosis in tumors treated with 5-FU and MCS three times a week for 2 weeks and found that the number of late apoptotic cells in the tumors increased with the combination of 5-FU and MCS (Fig. 4*E*). These results suggest that MCS enhances the p53-mediated apoptosis induced by 5-FU, contributing to its antitumor effect.

### Combination of MCS and 5-FU exhibits an antitumor effect against 5-FU-resistant MDA-MB-231 cells

**Figure 5:**
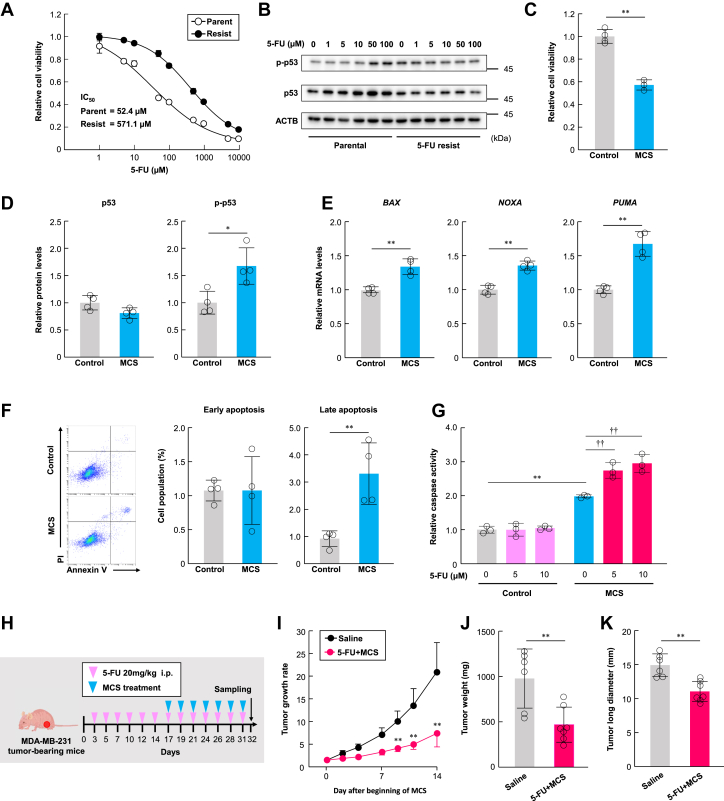
**Combination of MCS and 5-FU shows a high antitumor effect against 5-FU-resistant cells.***A*, Cell viability of parental and 5-FU-resistant MDA-MB-231 cells exposed to 5-FU. Cells were treated with 5-FU at concentrations ranging from 0 to 10000 μM for 72 h. The value for each 5-FU-untreated group is set at 1.0. Values are presented as the mean ± S.D. (n = 4). *B*, protein levels of p53 and phosphorylated p53 (p-p53) following treatment with 5-FU at concentrations ranging from 0 to 100 μM for 48 h. The uncropped image of p53 and p-p53 proteins in the cells is illustrated in Figure S13*D*. *C*, Effect of MCS treatment on cell viability in 5-FU-resistant MDA-MB-231 cells. The value for the non-MCS group is set at 1.0. Values are presented as the mean ± S.D. (n = 4). *D*, mRNA levels of p53 target pro-apoptotic genes *BAX, NOXA,* and *PUMA* in non-MCS-treated or MCS-treated 5-FU-resistant MDA-MB-231 cells. The value for the non-MCS group is set at 1.0. Values are presented as the mean ± S.D. (n = 4). ∗*p* < 0.05 indicates significant differences between the two groups (*t*_*6*_ = 5.535 for *BAX*; *t*_*6*_ = 7.074 for *PUMA*; Student’s *t* test). *E*, protein levels of p53, phosphorylated p53 (p-p53), and relative p53 phosphorylation levels in 5-FU-resistant MDA-MB-231 cells. The value for the non-MCS group is set at 1.0. Values are presented as the mean ± S.D. (n = 4). ∗∗*p* < 0.01 indicates a significant difference between the two groups (*t*_*6*_ = 3.416 for phosphorylated p53; *t*_*6*_ = 3.616 for relative p53 phosphorylation; Student’s *t* test). *F*, influence of MCS on apoptosis in 5-FU-resistant MDA-MB-231 cells. The *left panels* show flow cytometry analysis of annexin V in non-MCS-treated or MCS-treated 5-FU-resistant MDA-MB-231 cells. The *right panel* illustrates the difference in the ratio of FITC^+^/PI^-^ (early apoptosis) to FITC^+^/PI^+^ (late apoptosis) cell populations between non-MCS-treated or MCS-treated 5-FU-resistant MDA-MB-231 cells. The value for the non-MCS group is set at 1.0. Values are presented as the mean ± S.D. (n = 4). ∗∗*p* < 0.01 indicates a significant difference between the groups (*t*_*6*_ = 4.09 for late apoptosis; Student’s *t* test). *G*, caspase activity of parental and 5-FU-resistant MDA-MB-231 cells treated with 5-FU and MCS. The value of the non-MCS-treated without 5-FU treatment group is set at 1.0. Values are presented as the mean ± S.D. (n = 3). ∗∗*p* < 0.01 indicates a significant difference between the groups (*F*_*5,12*_ = 85.151, *p* < 0.001; ANOVA with the Tukey–Kramer *post hoc* test). ^††^*p* < 0.01 indicates a significant difference from the MCS-treated 5-FU 0 μM group (*F*_*2,6*_ = 18.866, *p* < 0.0026; ANOVA with Dunnett’s *post hoc* test). *H,* schematic representation of the experimental procedure for treating MDA-MB-231 transplanted mice with 5-FU alone for 2 weeks, followed by a combination of MCS and 5-FU for an additional 2 weeks 5-FU (20 mg/kg/mouse) was intraperitoneally administered to tumor-bearing mice three times a week. MCS treatment was performed after 5-FU administration. *I*, influence of combined MCS and 5-FU treatment on the tumor growth rate in MDA-MB-231-transplanted mice. Tumor volume at the start of the MCS treatment is set at 1.0. Values are presented as the mean ± S.D. (n = 6–7). ∗∗*p* < 0.01 indicates significant differences between the groups (*F*_*11,66*_ = 33.965, *p <* 0.001; ANOVA with a Tukey–Kramer *post hoc* test). *J*, tumor weight of MDA-MB-231 tumors treated with a combination of 5-FU and MCS for 2 weeks. Values are presented as the mean ± S.D. (n = 6–7). ∗∗*p* < 0.01 indicates a significant difference between the two groups (*t*_*11*_ = 3.476; Student’s *t* test). *K*, tumor long diameter of MDA-MB-231 tumors treated with a combination of 5-FU and MCS for 2 weeks. Values are presented as the mean ± S.D. (n = 4–7). ∗∗*p* < 0.01 indicates a significant difference between the two groups (*t*_*11*_ = 4.433; Student’s *t* test).

We investigated the combined effects of 5-FU and MCS on cells that had acquired resistance to 5-FU treatment. We attempted to generate 5-FU-resistant cells by exposing MDA-MB-231 cells to 5-FU (10 μM) for 2 weeks. The resulting cells exhibited notably increased resistance to 5-FU compared to the parent cells (Fig. 5*A*). We also evaluated the effect of 5-FU exposure on p53 in these cells and found that 48 h of 5-FU exposure did not induce p53 induction or phosphorylation (Fig. 5*B*). These results confirmed the establishment of 5-FU-resistant cells, prompting us to further evaluate the effect of MCS. First, we evaluated the effect of MCS on cell proliferation, both alone and in combination with 5-FU, and found that MCS inhibited cell proliferation in 5-FU-resistant cells (Fig. 5*C*).

The effects of MCS on p53-mediated induction of apoptotic genes and cell death were also evaluated. Increased p-p53 and expression of apoptotic genes were observed following MCS treatment (Fig. 5, *D* and *E*). Moreover, an increase in late apoptotic cells was observed with MCS (Fig. 5*F*). These results suggest that MCS is effective against MDA-MB-231 cells that have developed resistance to 5-FU. Therefore, we further evaluated the combined effects of 5-FU and MCS. *In vitro*, the combination of MCS improved the effect of 5-FU on caspase activity in 5-FU-resistant MDA-MB-231 cells (Fig. 5*G*). In addition, after 2 weeks of 5-FU administration, tumors were treated with the combination of 5-FU and MCS for a further 2 weeks (Fig. 5*H*). Two weeks of 5-FU alone accelerated tumor growth (Fig. 1, *D* and *E*); however, an additional 2 weeks of 5-FU administration and MCS treatment suppressed tumor growth (Fig. 5, *I*–*K*). The saline group was used as a control group for the 5-FU and MCS combination therapy group because most of the mice in the 5-FU monotherapy group reached the humane endpoint (tumor diameter: 20 mm, weight loss, tumor necrosis) around 3 weeks after the start of 5-FU administration. This is a limitation of this study, but it is possible to discuss the synergistic effect of 5-FU and MCS by comparing it with the saline group. Therefore, these results suggest that MCS is effective against tumors that have acquired resistance to 5-FU.

## Discussion

Chemotherapy for breast cancer involves a combination of anthracyclines, taxanes, and fluoropyrimidines, which are commonly used (1, 2). Among these, 5-FU efficacy is influenced by p53 status in carcinomas (5, 30). p53 mutations are frequently identified in breast cancer; however, the impact of p53 status on the therapeutic effect of 5-FU in breast cancer has not been fully elucidated. In the present study, 5-FU was less effective in breast cancer cells with p53 mutations. Conversely, MCS induced cell death in MDA-MB-231 cells by increasing intracellular metal ions and activating the mutant p53 transcriptional response. Furthermore, the combination of 5-FU and MCS showed high antitumor efficacy due to the induction of apoptosis *via* mutant p53, improving the efficacy of 5-FU. Based on these findings, we propose a therapeutic strategy incorporating MCS into breast cancer chemotherapy.

We confirmed the combined tumor-suppressive effect of 5-FU and MCS in tumor-bearing mice. However, there was an unexpected increase in MDA-MB-231 tumor growth after the administration of 5-FU alone (Fig. 1*C*). Previous studies have reported that 5-FU administration promotes the stemness of cancer cells *via* p53 and that MDA-MB-231 cells acquire stemness through p53 mutations (31, 32). Furthermore, it has been suggested that exposure to 5-FU induces the expression of *CD44*, a stem cell marker of breast cancer, in MDA-MB-231 cells (Fig. S7*A*) (33, 34). Additionally, aldehyde dehydrogenase (ALDH) activity, a marker of cancer stemness (35), was evaluated in MDA-MB-231 cell tumors exposed to 5-FU in combination with MCS. ALDH activity increased when 5-FU was administered alone, but this increase was suppressed when MCS was used in combination with 5-FU (Fig. S7*B*). These results suggest that 5-FU has a tumor-promoting effect on MDA-MB-231 cells *via* p53 carrying the R280K mutation and that MCS inhibits the stemness induced by 5-FU. Further analysis of p53-mediated mechanisms of these mutations remains warranted to optimize the 5-FU and MCS treatment strategy for cancer therapy.

Few studies have applied weak current stimulation to cancer cells. In this study, we found that MCS induced apoptosis in MDA-MB-231, MDA-MB-468, and MCF-7 cells (Fig. S8, *A*–*C*). Notably, in MDA-MB-231 cells, MCS-induced apoptosis was attributed to increased intracellular Cu^2+^ ions. Since MCS is a biological current, external electrical stimulation is known to alter the function of transporter proteins and ion channels (36, 37). Cu ions are most stable in their divalent form, and their uptake into cells primarily depends on the Cu^+^-selective transporter High Affinity Copper Uptake Protein 1 (CTR1) (38). CTR1 transports Cu^2+^, which is reduced to Cu^+^, across the cell membrane into the cell. However, some Cu^2+^ is also taken up in its divalent form by SLC11A2 (23). Chelating Cu^2+^ abolished the effects of MCS (Fig. S3, *A* and *B*). In contrast, the addition of the Cu^+^-selective chelating agent bathocuproinedisulfonic acid disodium salt did not affect the effect of MCS (Fig. S3*C*). Additionally, knocking out SLC11A2 abolished the effect of MCS (Fig. S4*A*). These results suggest that the effect of MCS is mediated by the influx of divalent metal ions *via* SLC11A2 rather than CTR1. The twofold difference in SLC11A2 expression levels between MDA-MB-231 and MCF-10A cells supports the breast cancer cell-specific effect of MCS (Fig. S4*B*). Furthermore, the findings of this study are reinforced by the observation that cancer cells enhance various transporter activities, including metal transport, for their own benefit (39, 40).

The results indicated that MCS increased Fe levels in MDA-MB-231 cells (Fig. 2*C*), and the loss of MCS's effect upon the knockout of *SLC11A2*, a Fe^2+^ uptake transporter (23) (Fig. S4*A*), suggests that Fe may play a role in the cytotoxic effect of MCS. Fe induces ferroptosis, a unique form of cell death mediated by lipid peroxidation. However, the absence of increased expression of ferroptosis-related genes, including *PTGS2* (41), in MDA-MB-231 cells after MCS treatment (Fig. S9, *A* and *B*), along with the lack of effect of ferrostatin, a ferroptosis inhibitor (42), on the cell proliferation inhibitory effect of MCS (Fig. S9*C*), indicates that Fe contributes little to the mechanism identified in this study. The high expression of GPX4, a potent ferroptosis-inhibitory enzyme, in MDA-MB-231 cells (43), coupled with the fact that the p53 pathway suppresses ferroptosis in TNBCs like MDA-MB-231 (44, 45), further supports the Fe-independent nature of the pathway identified in this study. Further studies using cell lines with a high susceptibility to ferroptosis may provide a more comprehensive understanding of the mechanism of MCS.

Exposure to EDTA or TTM, as well as the knockout of *SLC11A2*, suppressed the increase in p-p53 induced by MCS. However, these results also highlighted that Cu depletion increases p-p53 levels in MDA-MB-231 cells (Fig. S2*B*, S3*B*, and S4*A*). Copper is an essential cofactor in mitochondrial respiration and antioxidant pathways; thus, Cu depletion can cause various harmful effects in cells. In particular, copper/zinc superoxide dismutase (SOD1), which is responsible for ROS removal, requires Cu at its active site, and Cu deficiency can induce cell death (46). In fact, exposure to TTM reduced SOD activity in MDA-MB-231 cells (Fig. S10). These results suggest that the reduction in SOD activity associated with Cu depletion may increase p-p53 levels due to impaired ROS removal. Several pieces of evidence, including high intracellular Cu levels and SOD1 expression levels in many breast cancer cells, including MDA-MB-231 cells, as well as their protective effects on cells through ROS removal (47, 48), support the findings of the present study. The combination of SOD1 inhibitors, such as toferersen (49), may enhance the efficacy of MCS. On the other hand, the attempts to reduce the intracellular concentration of Cu in this study were indirect measures, such as using chelating agents and knockout, and it was not possible to eliminate Cu from the culture medium. This was due to the extreme difficulty of specifically removing Cu from the culture medium, particularly from FBS, and represents a limitation of this study. Technological innovations that enable this will likely contribute to a deeper understanding of intracellular Cu.

In this study, we evaluated the relationship between p53 mutation and efficacy, focusing on 5-FU, which is p53-dependent, and the effect of MCS-induced p53 activation on therapeutic efficacy. Besides these typical anticancer drugs, platinum agents are also sometimes used in treating metastatic breast cancer (50, 51). Like 5-FU, platinum drugs depend on p53 for their action (52, 53), and activation of the p53 pathway enhances their effects (54). Future studies should explore how MCS-induced p53 transcriptional activation affects anticancer drugs other than 5-FU, such as cisplatin, with a view toward clinical application.

In contrast to the *in vitro* results, MCS administration alone did not affect tumor volume (Fig. S11, *A* and *B*). However, in tumors, MCS increased caspase activity and decreased the number of Ki67-positive cells (Fig. S11, *C* and *D*), indicating induced apoptosis. These findings imply that MCS may also influence other cell types within the tumor microenvironment, such as fibroblasts and macrophages, as MCS has been shown to affect these cells (55, 56). Supporting this hypothesis, MCS increased the mRNA expression of the mouse-derived fibroblast marker *Col1a2* in tumors (Fig. S11*E*). These results suggest that MCS induces cancer cell apoptosis and fibroblast proliferation simultaneously, while no changes in tumor volume were observed. Further research on the effects of MCS on various cell types within the tumor microenvironment, including fibroblasts and immune cells, could provide insights for optimizing treatment.

We have previously investigated the effects of MCS on macrophages and found that MCS enhances macrophage phagocytosis by modulating their circadian clock (57). MCS-induced activation of macrophage phagocytosis also boosts acquired immunity through antigen presentation to T cells, resulting in the suppression of cancer cell engraftment and metastasis. In this study, MCS treatment was performed by applying an adhesive pad to cover the entire tumor implanted in nude mice (Fig. S12). Therefore, the effect of MCS on immune cells, such as macrophages, in the tumors of nude mice remains unclear. Nude mice are characterized by thymic defects, leading to T-cell maturation failure, and exhibit almost no acquired immune response. However, innate immunity remains intact, and the phagocytic ability of macrophages is activated (58, 59). The effect of MCS on tumors *via* macrophages in this unique immune environment remains unknown. Additionally, the effect of MCS on tumor-associated macrophages that infiltrate and accumulate in solid tumors is not yet clear. Therefore, it is imperative to further enhance our understanding by examining the effects of MCS on cancer immunity from various perspectives.

In breast cancer treatment, developing resistance to chemotherapy due to p53 mutations poses a major barrier, limiting treatment options. Additionally, chemotherapy side effects are problematic because anticancer agents also kill normal cells. These side effects reduce the quality of life for patients, making it crucial to achieve sufficient antitumor effects with lower chemotherapy doses. In this study, we demonstrated that short-term administration of MCS to breast cancer cells with p53 mutations not only induces cell death specifically in cancer cells but also enhances the efficacy of 5-FU, potentially providing adequate tumor growth suppression at low doses. The findings from our MCS studies are limited to those conducted in mice and cultured cells (17, 57). However, the goal of this study is to apply MCS to clinical treatment, and we anticipate that MCS will be utilized in various parts of the body. Therefore, the challenges of this study include verifying the efficacy of MCS in humans and other large animals and expanding its applicability to different body regions. Further analysis of the mechanisms and optimization of therapeutic stimulation conditions would contribute to safer and more effective breast cancer treatment methods in the future.

## Experimental procedures

### Cells and treatment

MDA-MB-231 human breast cancer cells (RRID: CVCL_0062), MDA-MB-468 human breast cancer cells (RRID: CVCL_0419), MCF-7 human breast cancer cells (RRID: CVCL_0031), and MCF-10A human breast cells (RRID: CVCL_0598) were purchased from American Type Culture Collection. MDA-MB-231 and MCF-7 cells were cultured in DMEM supplemented with 5% FBS (Moregte Biotech) and 0.5% penicillin–streptomycin solution (Invitrogen Life Technologies). MDA-MB-468 cells were cultured in DMEM/F12 (Gibco) supplemented with 5% FBS and 0.5% penicillin–streptomycin solution. MCF-10 A cells were cultured in DMEM/F12 supplemented with 5% FBS, 0.5% penicillin–streptomycin solution, 500 ng/ml hydrocortisone (Sigma-Aldrich), 10 μg/ml human recombinant insulin (FUJIFILM Wako Pure Chemical Corporation), and 20 ng/ml human recombinant epidermal growth factor (Funakoshi). Cells were maintained at 37 °C in a humidified 5% CO_2_ atmosphere. We confirmed the absence of microbes in these cell lines using a MycoBlue *Mycoplasma* Detector (Nanjing Vazyme Biotech).

Twenty-four hours before MCS, cells were seeded in 96-well plates. Cultured cells were exposed to 300 μA, 400 Hz bidirectional pulsed MCS for 30 min using ES-530 (Ito Co, Ltd) *via* platinum electrodes (Gold Shousha Co, Ltd), as described previously (17). The connection method and detailed current conditions are illustrated in Figure S12. Control cells underwent the same electrode placement and device connection but did not receive stimulation.

To downregulate the *p53* gene, *p53* shRNA (h) Lentivirus Particles (Santa Cruz Biotechnology) were transduced into MDA-MB-231 and MDA-MB-468 cells. The knockout of the *SLC11A2* gene in MDA-MB-231 cells was performed using the CRISPR/CRISPR-associated protein 9 system. Cells were transfected with *SLC11A2* CRISPR/CRISPR-associated protein 9 (Santa Cruz Biotechnology) and *SLC11A2* homology-directed repair (HDR) plasmids (Santa Cruz Biotechnology) using Lipofectamine LTX reagent (Thermo Fisher Scientific) according to the manufacturer’s instructions. After 24 h of incubation, cells were selected using 5 μg/ml puromycin. The medium was replaced with puromycin every few days for 2 weeks, after which the cells were isolated.

### Animals and treatment

All animal procedures adhered to the Animal Research: Reporting of In Vivo Experiments (ARRIVE) guidelines as well as the Guidelines for Animal Experiments of Kyushu University and received approval from the Institutional Animal Care and Use Committee of Kyushu University (protocol ID #A23–363–0, #A23–490–0). Five-week-old female Balb/c and Balb/c AJcl-nu/nu mice (Charles River Laboratory Japan, Inc) were housed under a standardized light–dark cycle at 24 ± 1 °C and 60 ± 10% humidity, with food and water provided ad libitum. MCF-7 (2 × 10^6^ cells), MDA-MB-231 (2 × 10^6^ cells), and MDA-MB-468 (5 × 10^6^ cells) suspended in 30 μl of PBS were implanted in the mammary fat pads of mice. Mice injected with cancer cells were intraperitoneally injected with 20 mg/kg 5-FU (FUJIFILM Wako Pure Chemical Corporation) three times a week. Tumor volume was estimated using the following formula:

Equation 1 $$\text{tumor}\;\text{volume}\;\left({\text{mm}}^{3}\right)=0.5\;\text{length}\;\left(\text{longest}\;\text{diameter}\right)\times \text{width}\;{\left(\text{shortest}\;\text{diameter}\right)}^{2}$$

MCS was conducted as previously described (17). Mice were anesthetized with isoflurane (Pfizer Inc), and electrode pads (Accelgard; PALS 879100, Φ32 mm × t1.3 mm) were attached to their abdomen and back. MCS-treated mice received 300 μA, 400 Hz bidirectional pulsed MCS for 30 min *via* a pad affixed to the ES-530 (Ito Co, Ltd). The structure of the MCS system for treating cells and mice is described in Figure S12.

### Cell viability assay

Cell viability after 5-FU exposure or MCS treatment was calculated from the amount of cellular ATP. Cells were seeded in 96-well plates at a density of 1.0 × 10^3^ cells/well. After 24 h of incubation, 5-FU exposure or MCS treatment was performed, and the amount of intracellular ATP was measured at each time point. The Cell Titer-Glo Luminescent Cell Viability Assay (Promega) was used to evaluate intracellular ATP, and chemiluminescence intensity was measured with an EnSpire Multimode Plate Reader (PerkinElmer).

### Establishment of 5-FU-resistant cells

5-FU-resistant cells were established from MDA-MB-231 by exposure to 5-FU. MDA-MB-231 cells were exposed to a 5-FU concentration of 10 μM in DMEM supplemented with 5% FBS for 2 weeks. The culture medium was replaced with fresh 5-FU-containing medium every 3 days. After exposure to 5-FU, cells were cultured in 5-FU-free medium for at least 3 days before use in each experiment.

### Measurement of metal ion content

Cells were lysed in RIPA buffer (20 mM Tris-HCl (pH 7.4), 150 mM NaCl, 0.1% SDS, 1% Nonidet P-40, deoxycholic acid, 2 mM EDTA), and equal amounts of nitric acid were added to the protein samples. Ion concentrations in the samples were measured using inductively coupled plasma mass spectrometry (ICP-MS; Agilent 7500c, 7700x; Agilent Technologies, Ltd).

### Measurement of ROS levels

Cells were seeded in 96-well plates at 5.0 × 10^3^ cells/well, incubated for 24 h, and then electrically stimulated with a platinum electrode. After stimulation for 30 min, intracellular ROS levels were measured using an ROS assay kit with the highly sensitive DCFH-DA reagent (Dojindo Laboratories) following the manufacturer’s protocol. Fluorescence intensity was measured quantitatively using a computer-assisted image analyzer under a BZ-X800 fluorescence microscope (Keyence).

### Construction of cell lines stably expressing luciferase reporter vectors

The 992 and 204 bp promoter regions of *BAX* were cloned into the pGL4.18 [*luc2P*/Neo] Luciferase plasmid (Promega, #E6731). The primer information used for cloning is listed in Table S1. Each vector was transfected using Lipofectamine LTX reagent according to the manufacturer’s instructions. After 24 h of incubation, cells were selected using 4000 μg/ml G418 (FUJIFILM Wako Pure Chemical Corporation) for 1 week to create stable expression lines.

### Luciferase reporter assay

Cells stably expressing a luciferase reporter vector were seeded in 96-well plates at 2.5 × 10^4^ cells/well, incubated for 24 h, and electrically stimulated with a platinum electrode. At 24 h after stimulation, luciferase activity was analyzed using the Bright-Glo Luciferase Assay System (Promega).

### Immunohistochemical staining of Ki-67

To assess tumor cell proliferation, immunohistochemical staining was performed using a monoclonal antibody against Ki-67 (Agilent, Cat# M7240, RRID: AB_2142367). Tumor tissues were removed from mice and fixed with 4% paraformaldehyde in PBS 6 weeks after implantation. Paraffin-embedded tumor sections were deparaffinized and rehydrated through graded ethanol, followed by the blocking of endogenous peroxidase. Tumor sections were then subjected to heat-induced antigen retrieval and incubated with the primary antibody. Specific antigen–antibody complexes were visualized using horseradish peroxidase–conjugated secondary antibodies with a 3,3′-diaminobenzidine (DAB) solution, and sections were counterstained with hematoxylin. The DAB staining regions were measured quantitatively using a computer-assisted image analyzer under a Keyence BZ-X800 fluorescence microscope.

### Isolation of tumor-derived cells

Two or 4 weeks after the start of MCS treatment, mouse hearts were exposed and perfused with 10 ml PBS from the left ventricle. Removed tumors were digested in PBS containing 500 μg/ml collagenase (FUJIFILM Wako Pure Chemical Corporation), 200 μg/ml CaCl_2_, 0.05% trypsin, and 10% FBS at 37 °C for 10 min under agitation. Digested samples were passed through a 23-gauge needle three times for further dissociation. Red blood cells were then lysed in Red Blood Lysis buffer (BioLegend), and cells were filtered through a 40-μm strainer to obtain a cell suspension.

## Flow cytometry

### Annexin V apoptosis assay

Dissociated single cells from cell lines or tumor specimens were suspended in a mixture of binding buffer, Annexin-FITC, and propidium iodide (MBL) and then incubated at 25 °C for 15 min. Cell separation was performed on a BD FACSAria III Cell Sorter (Becton Dickinson Biosciences) with FACSDiva software v6.1.3 and analyzed using FlowJo software version 10.5.3 (Becton Dickinson Biosciences).

### ALDEFLUOR assay

Dissociated single cells from cell lines were suspended in ALDEFLUOR assay buffer containing an ALDH substrate (BAAA) at 1.5 μM and incubated for 40 min at 37 °C. A specific inhibitor of ALDH, diethylamino benzaldehyde (DEAB), at a 10-fold molar excess, was used as a negative control. Fluorescence-activated cell detection was performed on a BD FACSAria III Cell Sorter (Becton Dickinson Biosciences) with FACSDiva software v6.1.3 and analyzed using FlowJo software version 10.5.3 (Becton Dickinson Biosciences).

### Determination of caspase-3/7 activity

Cultured cells were seeded in 96-well plates at 5.0 × 10^3^ cells/well, incubated for 24 h, and then electrically stimulated with a platinum electrode. At 24 h after stimulation, caspase-3/7 activity was measured using a Caspase-Glo 3/7 Assay Kit (Promega). Tumor specimens were lysed in CelLytic MT Cell Lysis Reagent (Sigma-Aldrich), and the resulting protein extracts were used to evaluate caspase-3/7 activity. Caspase-3/7 activity was determined using the Apo-One caspase-3/7 reagent kit (Promega).

### Quantitative RT-PCR

Total RNA was extracted using the ReliaPrep RNA Mini Prep System (Promega) or RNAiso (Takara Bio Inc), following the manufacturer's instructions. cDNA was synthesized using the ReverTra Ace qPCR RT Kit (Toyobo Life Science). Real-time PCR analysis was performed on diluted cDNA samples utilizing the THUNDERBIRD SYBR qPCR Mix (Toyobo Life Science) with the 7500 Real-Time PCR System (Applied Biosystems). Data were normalized against 18S and β-actin mRNA as internal controls. Primer sequences are listed in Table S2.

### Western blotting

Total protein was extracted using CelLytic MT Cell Lysis Reagent (Sigma-Aldrich). The protein extracts were mixed with 2 × sample buffer and denatured at 95 °C for 5 min. Samples were then separated by SDS-PAGE and transferred to a polyvinylidene difluoride membrane. Membranes were reacted with horseradish peroxidase-conjugated antibodies against p53 (Cell Signaling Technology, Cat# 2524, RRID: AB_331743), phospho-p53 (Ser15) (Cell Signaling Technology, Cat# 9284, RRID: AB_331464), SLC11A2 (Abcam, Cat# ab55735), and β-actin (Santa Cruz Biotechnology, Cat# sc-47778, RRID: AB_626632). Immunocomplexes were detected using anti-mouse (Abcam, Cat# ab205719, RRID: AB_2755049) or anti-rabbit IgG (Abcam, Cat# ab205718, RRID: AB_2819160) secondary antibodies, and signals were visualized with ImmunoStar LD (FUJIFILM Wako Pure Chemical Corporation). Images were captured and analyzed using FUSION FX (M&S Instruments Inc).

### ChIP analysis

ChIP assays were conducted using the SimpleChIP Enzymatic Chromatin IP Kit (Cell Signaling Technology) with antibodies targeting p53 (Cell Signaling Technology, Cat# 2524, RRID: AB_331743). DNA was amplified using PCR, and the products were separated by electrophoresis. PCR products were stained with ethidium bromide, and the staining intensity was quantified. All data were normalized to the PCR products of input DNA. Primer sequences for amplification are provided in Table S3.

### Measurement of SOD activity

SOD activity of cells were measured using an SOD assay kit-WST (Dojindo) following the manufacturer’s protocol. SOD active units were defined from the SOD inhibitory activity of each sample by the WST method.

### RNA sequencing

Total RNA was extracted from cells using the ReliaPrep RNA Miniprep System (Promega). For RNA sequencing, sequencing libraries were prepared from 200 ng of total RNA using the MGIEasy rRNA Depletion Kit and MGIEasy RNA Directional Library Prep Set (MGI Tech Co, Ltd) according to the manufacturer's instructions. Libraries were sequenced on the DNBSEQ-G400 FAST Sequencer (MGI Tech Co, Ltd) using a paired-end 150 nt strategy. For sequencing data analysis, all sequencing reads were trimmed to remove low-quality bases and adapters using Trimmomatic (v.0.38) (60). Raw counts for each gene were estimated in each sample using RSEM version 1.3.0 and Bowtie 2 (61, 62). Differentially expressed genes (DEGs) were identified using the edgeR (63) program. Normalized counts per million (CPM) values and log fold-changes (log_2_FC) were derived from gene-level raw counts. Criteria for DEGs were established as *p*-value ≤ 0.05 and log_2_FC ≥ 1 for upregulated genes.

### Statistical and data analyses

Statistical analyses were performed using JMP software version 16 (SAS Institute). Values are expressed as the mean ± SD. All experiments were performed at least in triplicate. The significance of differences between two groups was analyzed using two-tailed Student’s t-tests, while those with more than two groups were determined through analysis of variance (ANOVA) followed by Tukey–Kramer *post hoc* tests or Dunnett’s test. Equal variances were not formally tested. A 5% level of probability was considered significant.

## Data availability

All data supporting the results of this study are included in the article, either in the main figures or the Supporting Information files. RNA sequencing data generated in this study were submitted to the Gene Expression Omnibus (GEO) at the National Center for Biotechnology Information (MCS-treated MDA-MB-231 cells: GSE255313). Functional analyses of the RNA sequencing datasets were performed using the Gene Ontology Resource (http://geneontology.org/). Any additional data supporting the findings of this study will be available from the corresponding author upon reasonable request.

## Supporting information

This article contains supporting information.

## Conflict of interest

The authors declare that they have no conflicts of interest with the contents of this article.
