# Supplementary material for: Microcurrent stimulation induces cell death in p53-mutant and 5-FU-resistant breast cancer
Source: J Biol Chem. 2025 Jun 24;301(8):110414. doi: 10.1016/j.jbc.2025.110414 (PMC12301737; doi:10.1016/j.jbc.2025.110414)
Supplement: Supporting information [file mmc1.docx]

**Supporting information**

**Microcurrent stimulation induces cell death in p53-mutant and 5-FU-resistant breast cancer**

Tomohito Tanihara, Yuya Yoshida, Takashi Ogino, Yuma Terada, Fumiaki Tsurusaki, Keika Hamasaki, Kaita Otsuki, Kohei Fukuoka, Kosuke Oyama, Akito Tsuruta, Kengo Hamamura, Kouta Mayanagi, Satoru Koyanagi, Yuichi Murakami, Mayumi Ono, Michihiko Kuwano, Shigehiro Ohdo^*^, Naoya Matsunaga^*^

*Corresponding authors:

Shigehiro Ohdo

Department of Pharmaceutics, Faculty of Pharmaceutical Sciences, Kyushu University, 3-1-1 Maidashi Higashi-ku, Fukuoka 812-8582, Japan

Email: [ohdo@phar.kyushu-u.ac.jp](mailto:ohdo@phar.kyushu-u.ac.jp); Telephone: 08-092-642-6610

Naoya Matsunaga

Department of Clinical Pharmacokinetics, Faculty of Pharmaceutical Sciences, Kyushu University, 3-1-1 Maidashi Higashi-ku, Fukuoka 812-8582, Japan

Email: [matunaga@phar.kyushu-u.ac.jp](mailto:matunaga@phar.kyushu-u.ac.jp); Telephone: 08-092-642-6656

**This file includes:**

Figures S1 to S13

Tables S1 to S3

**
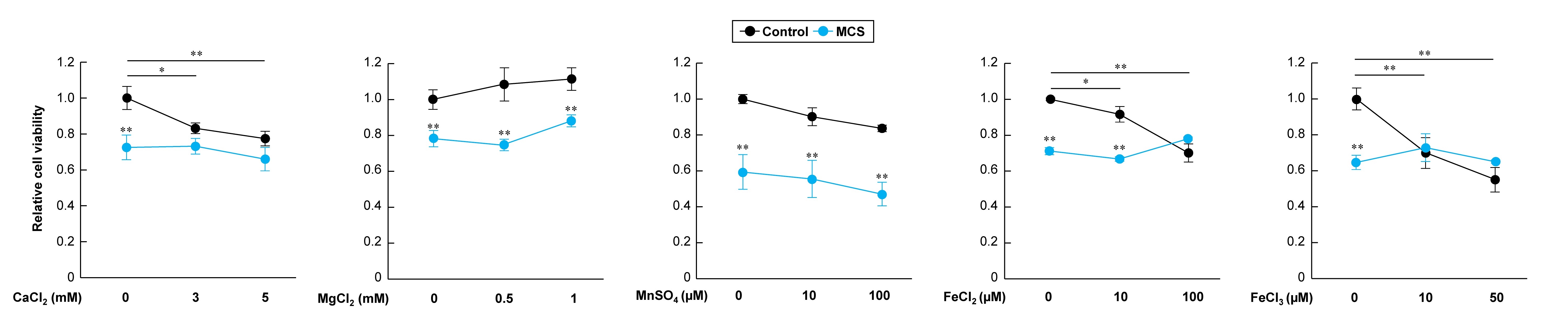
**

**Figure S1. Effect of the addition of metal ions to the medium.**

The effect of adding metal ions to the medium on cell viability after MCS treatment in MDA-MB-231 cells. Cell viability was assessed 48 hours after MCS treatment in the presence of metal ions. Values are presented as the mean ± S.D. (n = 3), with the control group (0 µM metal ions) set to 1.0. **P* < 0.05 and ***P* < 0.01 indicate significant differences from the control group. The statistical analysis results are as follows: *F_5,12_* = 14.502, *P* < 0.001 for CaCl_2_; *F_5,12_* = 21.975, *P* < 0.001 for MgCl_2_; *F_5,12_* = 29.661, *P* < 0.001 for MnSO_4_; *F_5,12_* = 63.721, *P* < 0.001 for FeCl_2_; and *F_5,12_* = 17.977, *P* < 0.001 for FeCl_3_ (ANOVA with Tukey–Kramer post-hoc test).

**
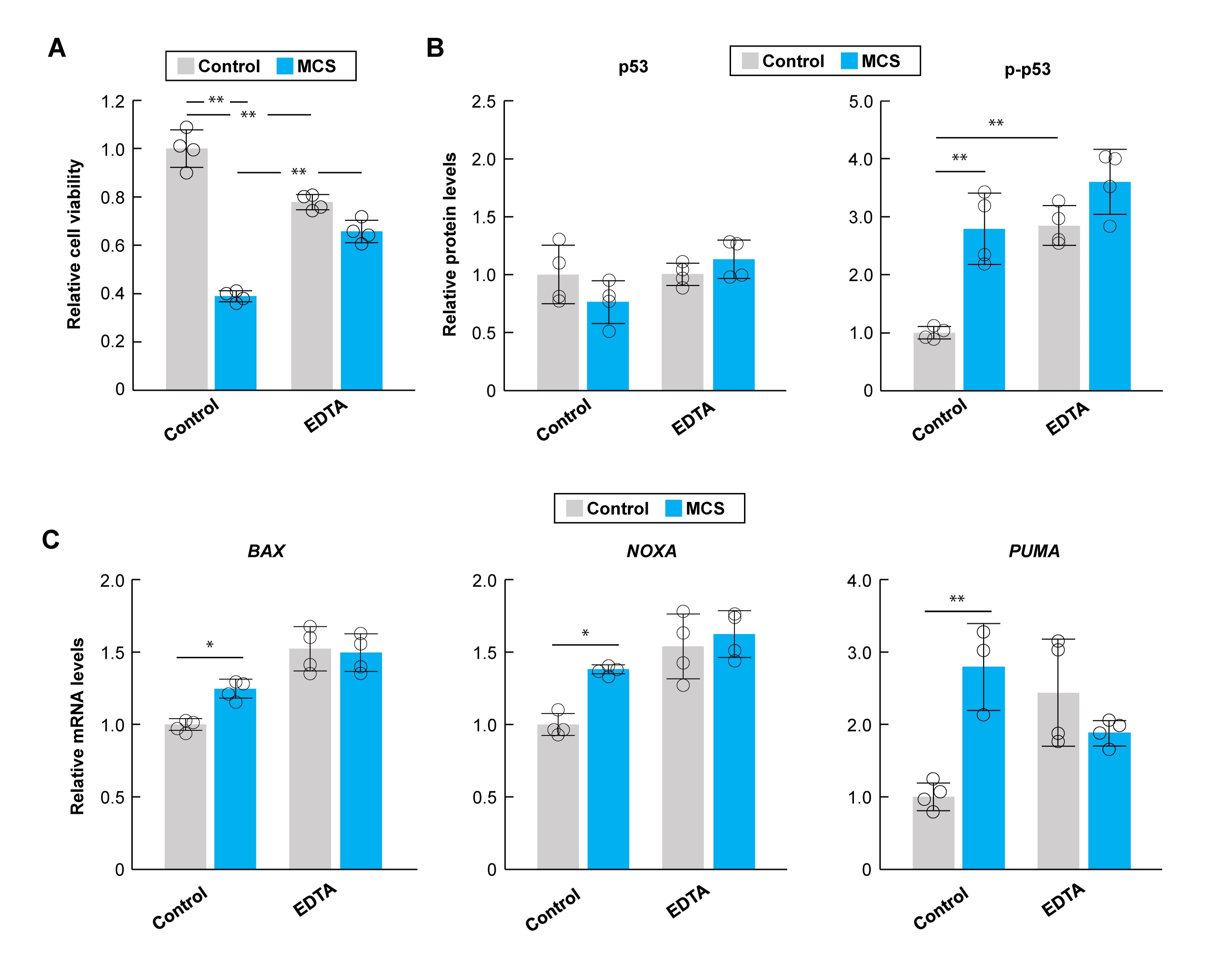
**

**Figure S2. Influence of EDTA on the efficacy of MCS.**

**(A)** Influence of EDTA on the inhibition of MDA-MB-231 cell proliferation induced by MCS. Cell viability was assessed 48 hours after MCS treatment in the presence of EDTA (1 mM). The value of the non-MCS-treated without EDTA group is set at 1.0. Values are presented as the mean ± S.D. (n = 4). ^**^*P* < 0.01 indicates significant differences between the two groups (*F_3,12_* = 113.016, *P* < 0.001; ANOVA with a Tukey–Kramer post-hoc test). **(B)** Influence of EDTA on the protein levels of p53 and p-p53 in MDA-MB-231 cells. The value of the non-MCS-treated without EDTA group is set at 1.0. Values are presented as the mean ± S.D. (n = 4). ***P* < 0.01 indicates a significant difference between the groups (*F_3,12_* = 23.885, *P* < 0.001 for p-p53; ANOVA with a Tukey–Kramer post-hoc test).(**C)** Effect of EDTA on MCS-induced pro-apoptotic gene transcription in MDA-MB-231 cells. The value of the non-MCS-treated without EDTA group is set at 1.0. Values are presented as the mean ± S.D. (n = 3–4). **P* < 0.05 and ***P* < 0.01 indicate significant differences between the groups (*F*_3,12_ = 20.741, *P* < 0.001 for *BAX*; *F*_3,12_ = 14.604, *P* = 0.0003 for *NOXA*; *F*_3,11_ = 9.617, *P* = 0.0021 for *PUMA*; ANOVA with a Tukey–Kramer post-hoc test).

**
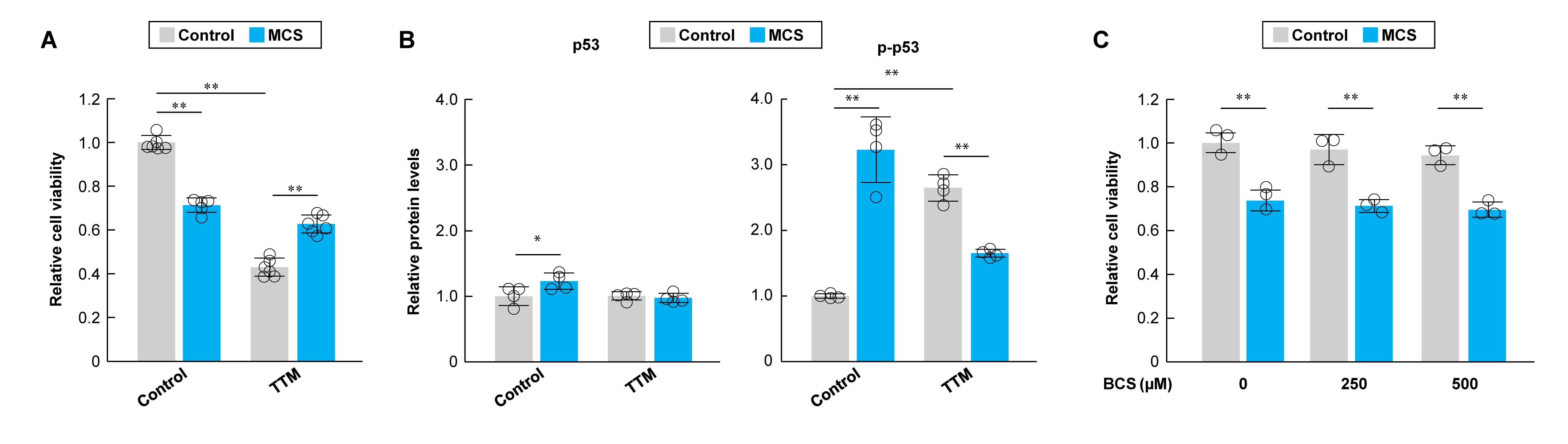
**

**Figure S3. Influence of TTM on the efficacy of MCS.**

**(A)** Effect of TTM (10 µM) on the inhibition of MDA-MB-231 cell proliferation induced by MCS. Cell viability was assessed 48 hours after MCS treatment in the presence of TTM. The value of the non-MCS-treated without TTM group is set at 1.0. Values are presented as the mean ± S.D. (n = 4). ^**^*P* < 0.01 indicates significant differences between the two groups (*F_3,19_* = 241.15, *P* < 0.001; ANOVA with a Tukey–Kramer post-hoc test). **(B)** Effect of TTM on the protein levels of p53 and p-p53 in MDA-MB-231 cells. The value of the non-MCS-treated without TTM group is set at 1.0. Values are presented as the mean ± S.D. (n = 4). ***P* < 0.01 indicates a significant difference between the groups (*F_3,12_* = 5.115, *P* = 0.017 for p53; *F_3,12_* = 53.599, *P* < 0.001 for p-p53; ANOVA with a Tukey–Kramer post-hoc test). **(C)** Effect of bathocuproinedisulfonic acid (BCS) on the inhibition of MDA-MB-231 cell proliferation induced by MCS. Cell viability was assessed 48 hours after MCS treatment in the presence of BCS. The value of the non-MCS-treated without BCS group is set at 1.0. Values are presented as the mean ± S.D. (n = 3). ^**^*P* < 0.01 indicates significant differences between the two groups (*F_5,12_* = 24.633, *P* < 0.001; ANOVA with a Tukey–Kramer post-hoc test).


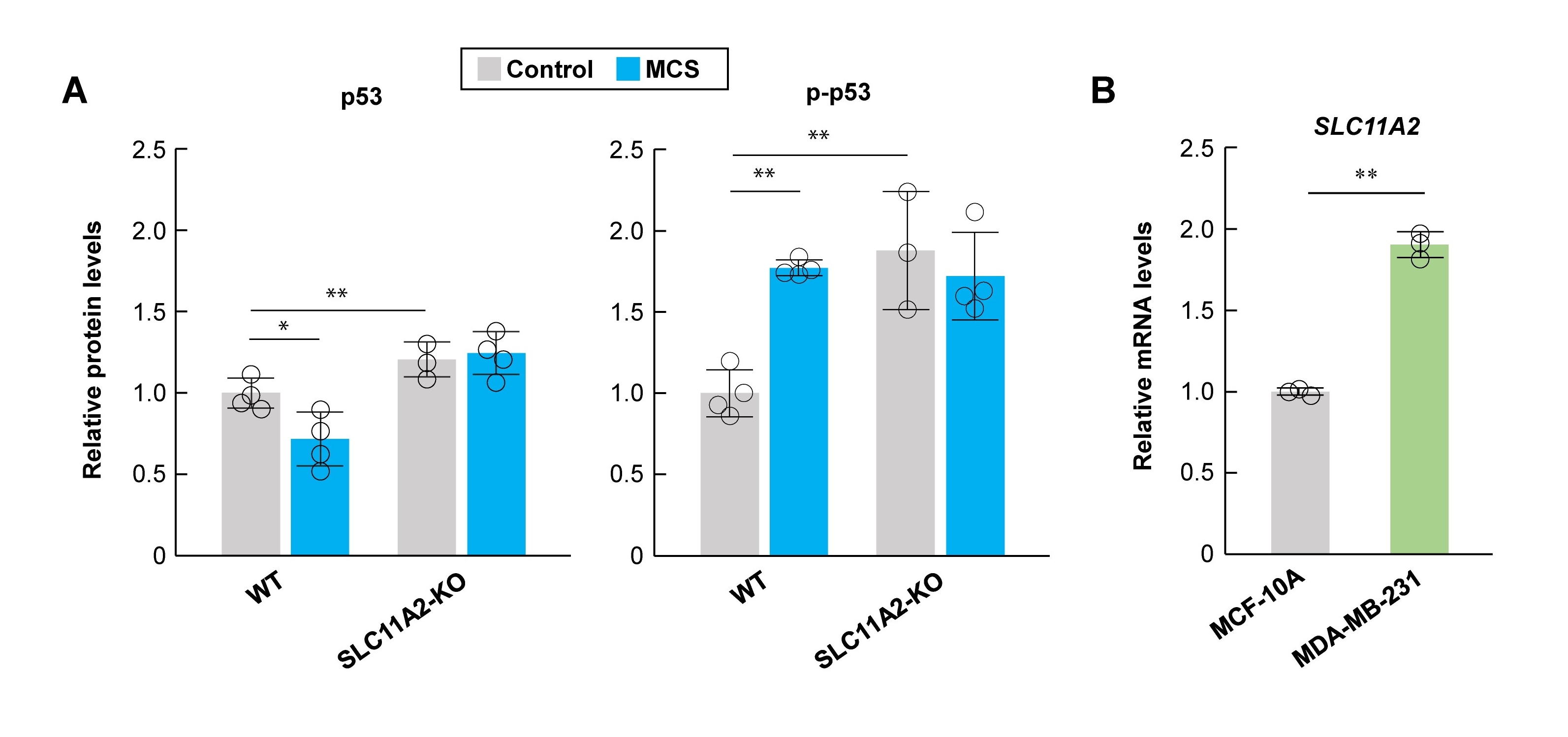


**Figure S4. Relationship between *SLC11A2* expression and the effects of MCS.**

**(A)** Influence of the *SLC11A2* knockout on the protein levels of p53 and p-p53 after MCS treatment in MDA-MB-231 cells. The value for the WT non-MCS-treated group is set at 1.0. Values are presented as the mean ± S.D. (n = 3–4). **P* < 0.05 and ***P* < 0.01 indicate significant differences between the groups (*F_3,11_* = 13.46, *P* < 0.001 for p53; *F_3,11_* = 12.175, *P* < 0.001 for p-p53; ANOVA with a Tukey–Kramer post-hoc test). The expression of the SLC11A2 protein in the cells is illustrated in **Figure S13F**. **(B)** mRNA levels of *SLC11A2* in MCF-10A and MDA-MB-231 cells. The value for MCF-10A cells is set at 1.0. Values are presented as the mean ± S.D. (n = 3). ***P* < 0.01 indicates significant differences between the two groups (*t_4_* = 19.099; Student’s t-test).

**
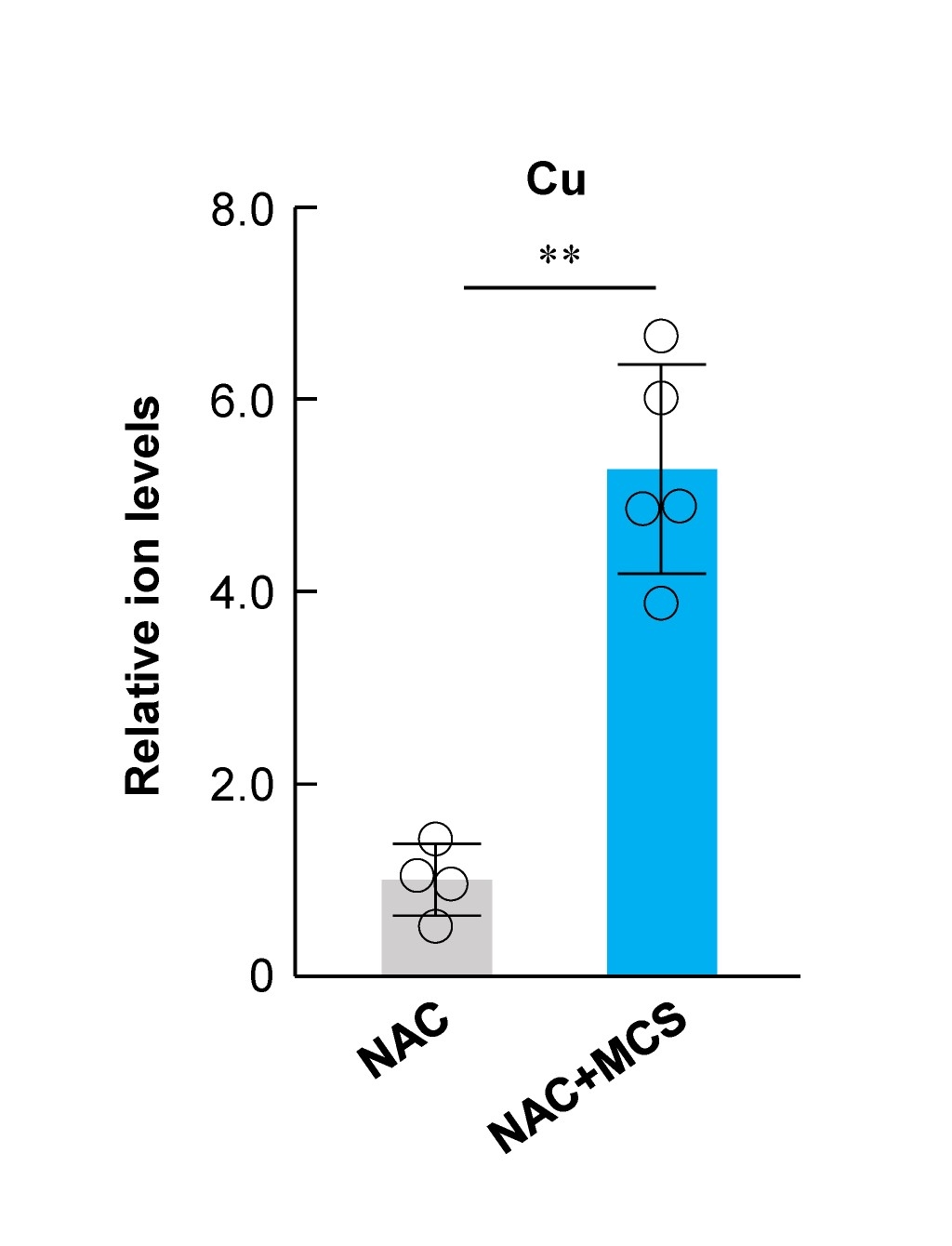
**

**Figure S5.** Influence **of NAC on copper ion influx by MCS.**

Effect of NAC (5 mM) on MCS-induced copper ion influx in MDA-MB-231 cells. Cell viability was assessed 48 hours after MCS treatment in the presence of NAC. The NAC group value is set at 1.0. Values are presented as the mean ± S.D. (n = 4–5). ^**^*P* < 0.01 indicates significant differences between the two groups (*t_7_* = 7.432; Student’s t-test).


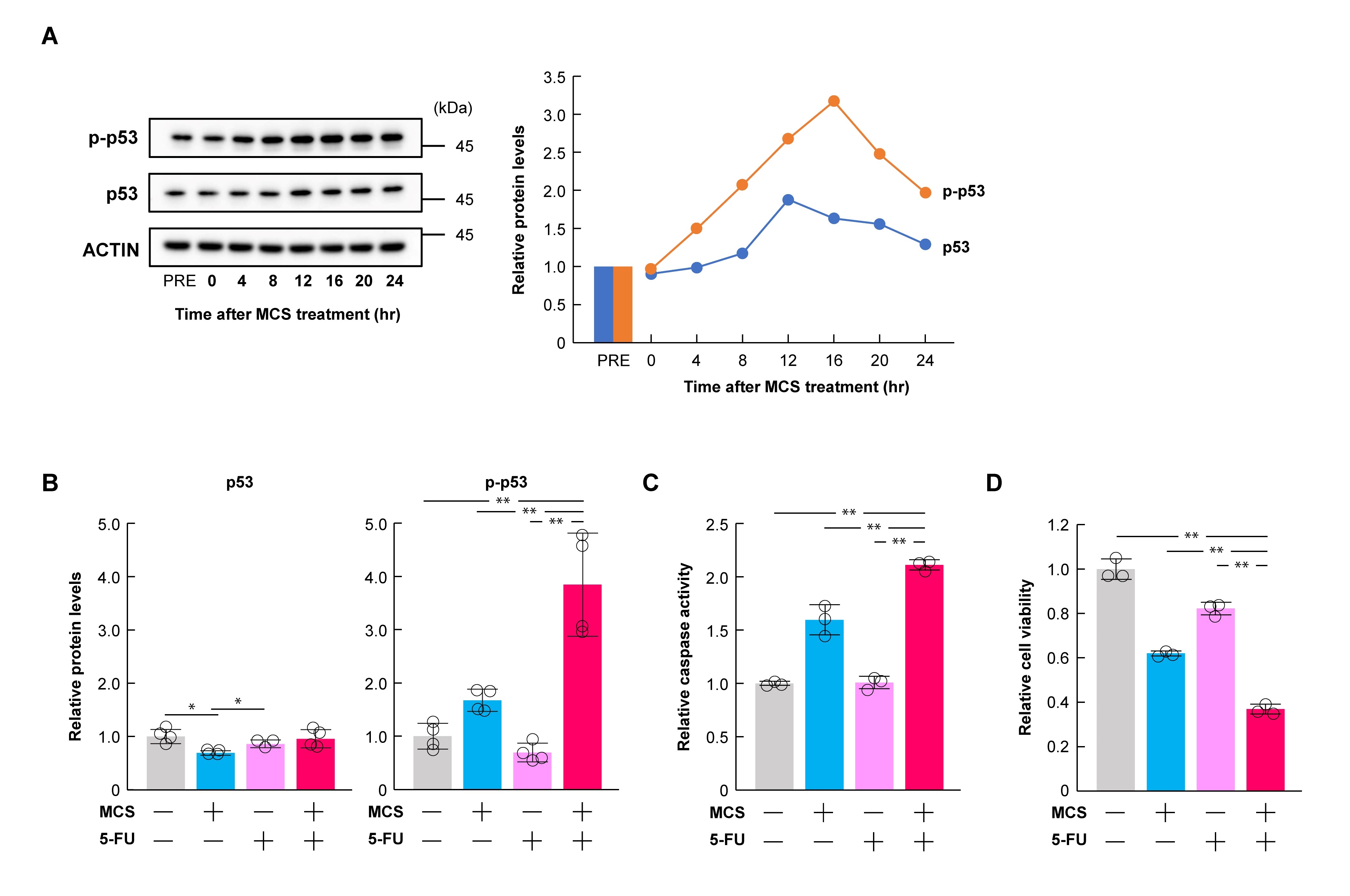


**Figure S6. Effect of MCS pre-treatment on the efficacy of 5-FU.**

**(A)** Time course of p53 and p-p53 protein levels measured every 4 hours after MCS treatment in MDA-MB-231 cells. The value for the pre-MCS-treated group is set at 1.0. An uncropped image of the p53 protein in the cells is illustrated in **Figure S13E**. **(B)** Protein levels of p53, phosphorylated p53 (p-p53) levels in MDA-MB-231 cells exposed to 5-FU (10 µM) after MCS pre-treatment. The 5-FU-untreated non-MCS group is set at 1.0. ***P* < 0.01 and **P* < 0.05 indicates a significant difference between the groups (*F_3,11_* = 5.245, *P* = 0.0172 for p53; *F_3,12_* = 30.204, *P* < 0.001 for p-p53; ANOVA with a Tukey–Kramer post-hoc test). **(C)** Caspase activity in MDA-MB-231 cells exposed to 5-FU after MCS pre-treatment. The 5-FU-untreated non-MCS group is set at 1.0. Values are presented as the mean ± S.D. (n = 3). ***P* < 0.01 indicates a significant difference between the groups (*F_5,12_* = 62.821, *P* < 0.001; ANOVA with the Tukey–Kramer post-hoc test). ^†^*P* < 0.05 indicates a significant difference from the MCS-treated 5-FU 0 µM group (*F_2,6_* = 5.952, *P* < 0.0376; ANOVA with Dunnett’s post-hoc test). **(D)** Cell viability in MDA-MB-231 cells exposed to 5-FU (10 µM) after MCS pre-treatment, assessed 48 hours after MCS treatment. The 5-FU-untreated non-MCS group is set at 1.0. Values are presented as the mean ± S.D. (n = 3). ***P* < 0.01 indicates a significant difference between the groups (*F_3,8_* = 62.821, *P* < 0.001; ANOVA with the Tukey–Kramer post-hoc test).


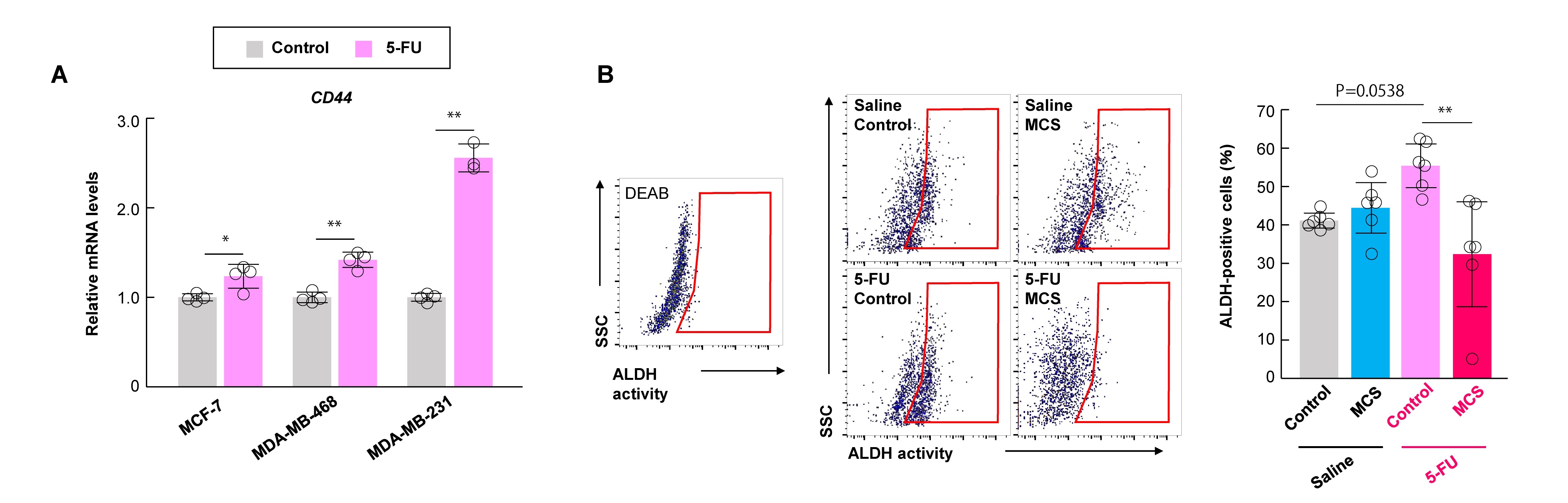


**Figure S7. Effect of 5-FU and MCS combination treatment on ALDH activity in tumors.**

**(A)** mRNA levels of *CD44* in breast cancer cells exposed to 5-FU (10 µM) for 48 h. The non-MCS treated group value is set at 1.0. Values are presented as the mean ± S.D. (n = 3–4). **P* < 0.05 and ***P* < 0.01 indicate significant differences from each control (MCF-7; *t*_6_ = 3.371 for *CD44*; MDA-MB-468; *t*_6_ = 7.991 for *CD44*; MDA-MB-231; *t*_5_ = 19.571 for *CD44*; Student’s t-test). **(B)** ADEFLUOR assay of MDA-MB-231 tumor cells in mice treated with a combination of MCS and 5-FU. The population of ALDH-positive cells was defined based on each DEAB group, which exhibited nearly identical mean fluorescence intensity values. The right panel displays the differences in ALDH-positive cells within the MDA-MB-231 tumors of each mouse group. Values are presented as the mean ± S.D. (n = 6). ***P* < 0.01 indicates a significant difference between the groups (*F_3,20_* = 6.814, *P* = 0.0024; ANOVA with a Tukey–Kramer post-hoc test).

**
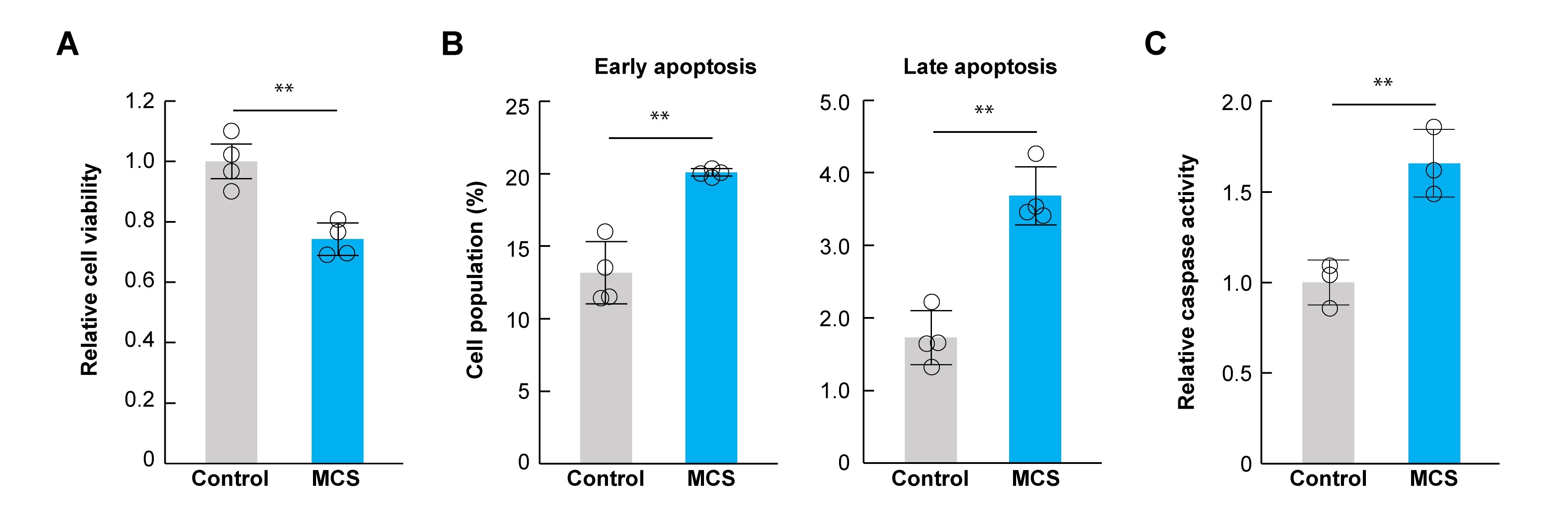
**

**Figure S8. Effect of MCS on MCF-7 cells.**

**(A)** Cell viability in MCF-7 cells 48 hours after MCS treatment. The value for each control group is set at 1.0. Values are presented as the mean ± S.D. (n = 4). ***P* < 0.01 indicates significant differences between the two groups (*t_6_* = 5.054; Student’s t-test). **(B)** Flow cytometry analysis of Annexin-FITC⁺/PI⁻ (early apoptosis) and Annexin-FITC⁺/PI⁺ (late apoptosis) cell populations in MCF-7 cells 24 hours after MCS treatment. The value for each control group is set at 1.0. Values are presented as the mean ± S.D. (n = 4). ***P* < 0.01 indicates significant differences between the two groups (*t_6_* = 6.391 for early apoptosis; *t_6_* = 7.14 for late apoptosis; Student’s t-test). **(C)** Caspase-3/7 activity in MCS-treated MCF-7 cells 24 hours after MCS treatment. The value for each control group is set at 1.0. Values are presented as the mean ± S.D. (n = 3). ***P* < 0.01 indicates significant differences between the two groups (*t_4_* = 5.088; Student’s t-test).


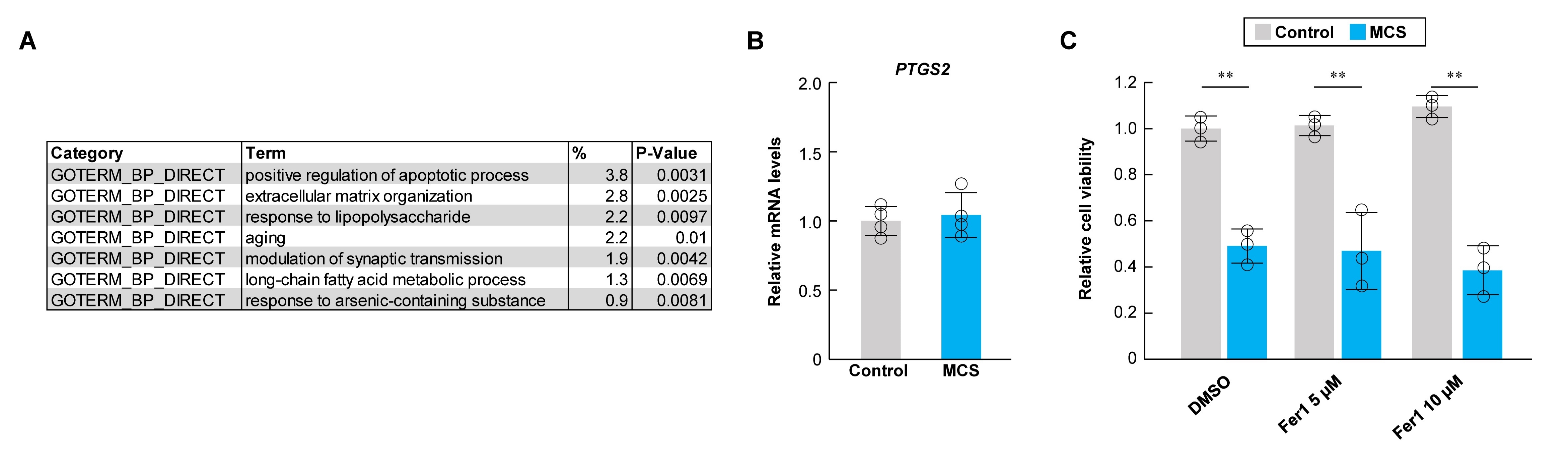


**Figure S9. Effect of MCS on ferroptosis in MDA-MB-231 cells.**

**(A)** Gene Ontology analysis of genes showing MCS-dependent expression variation based on RNA-seq results in MDA-MB-231 cells 24 hours after MCS treatment. The analysis includes genes with a |MCS / non-MCS ratio| > 2, and the terms are filtered for *P* < 0.01**.** The term list is sorted by percentage. **(B)** mRNA levels of the ferroptosis marker *PTGS2* in MDA-MB-231 cells treated with MCS. The value for the non-MCS-treated group is set at 1.0. Values are presented as the mean ± S.D. (n = 4). **(C)** Effect of ferrostatin (Fer1), a ferroptosis inhibitor, on cell viability following MCS treatment in MDA-MB-231 cells. Values are presented as the mean ± S.D. (n = 3), with the value for the DMSO non-MCS-treated group set at 1.0. **P* < 0.05 and ***P* < 0.01 indicate significant differences from each control (*F_5,12_* = 18.715, *P* < 0.001; ANOVA with a Tukey–Kramer post-hoc test).

**
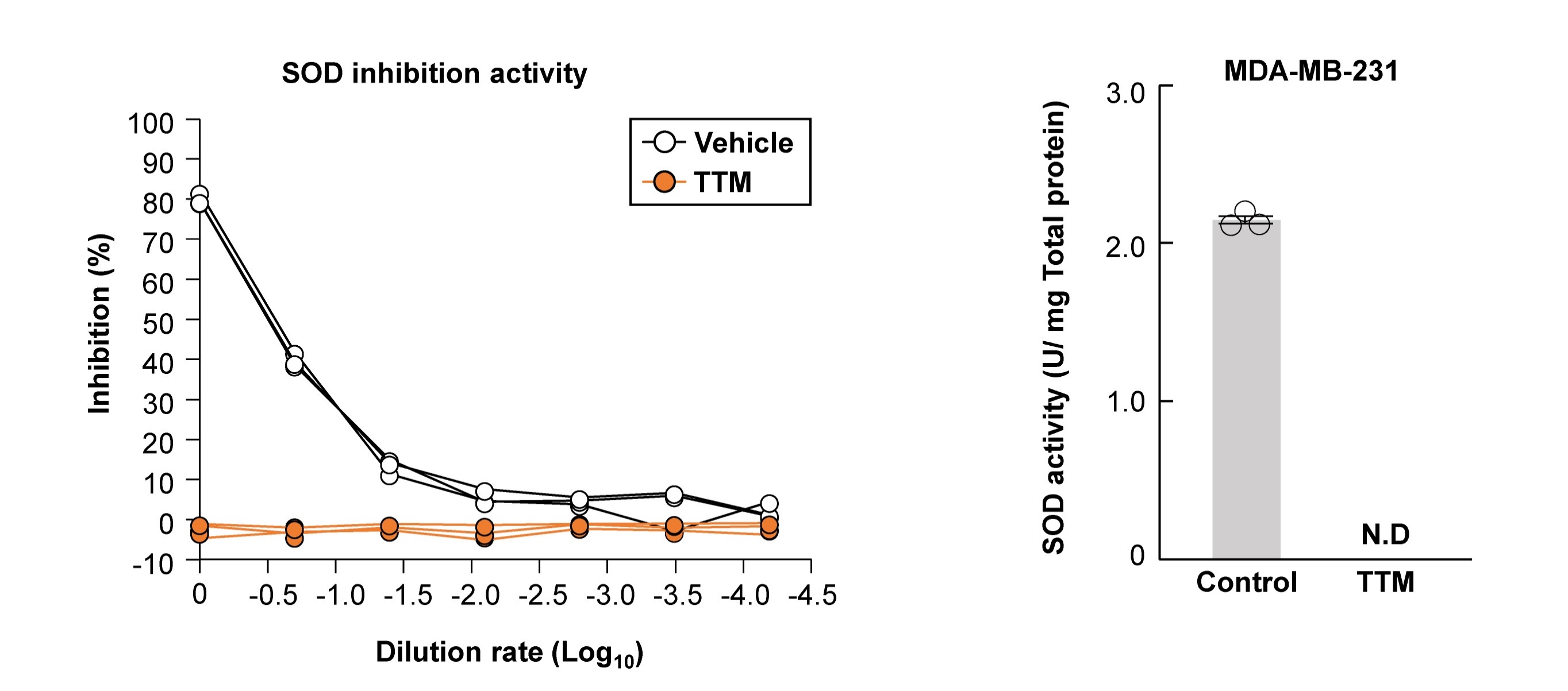
**

**Figure S10. Copper deficiency and SOD activity in MDA-MB-231 cells**

Effect of TTM on SOD activity in MDA-MB-231 cells. Left panel shows SOD inhibitory activity curve of extracts from MDA-MB-231 cells exposed to TTM. Right panel shows the number of units defined by the WST method calculated from the SOD inhibitory activity curve. Values are presented as the mean ± S.D. (n = 3). N.D: Not detected.


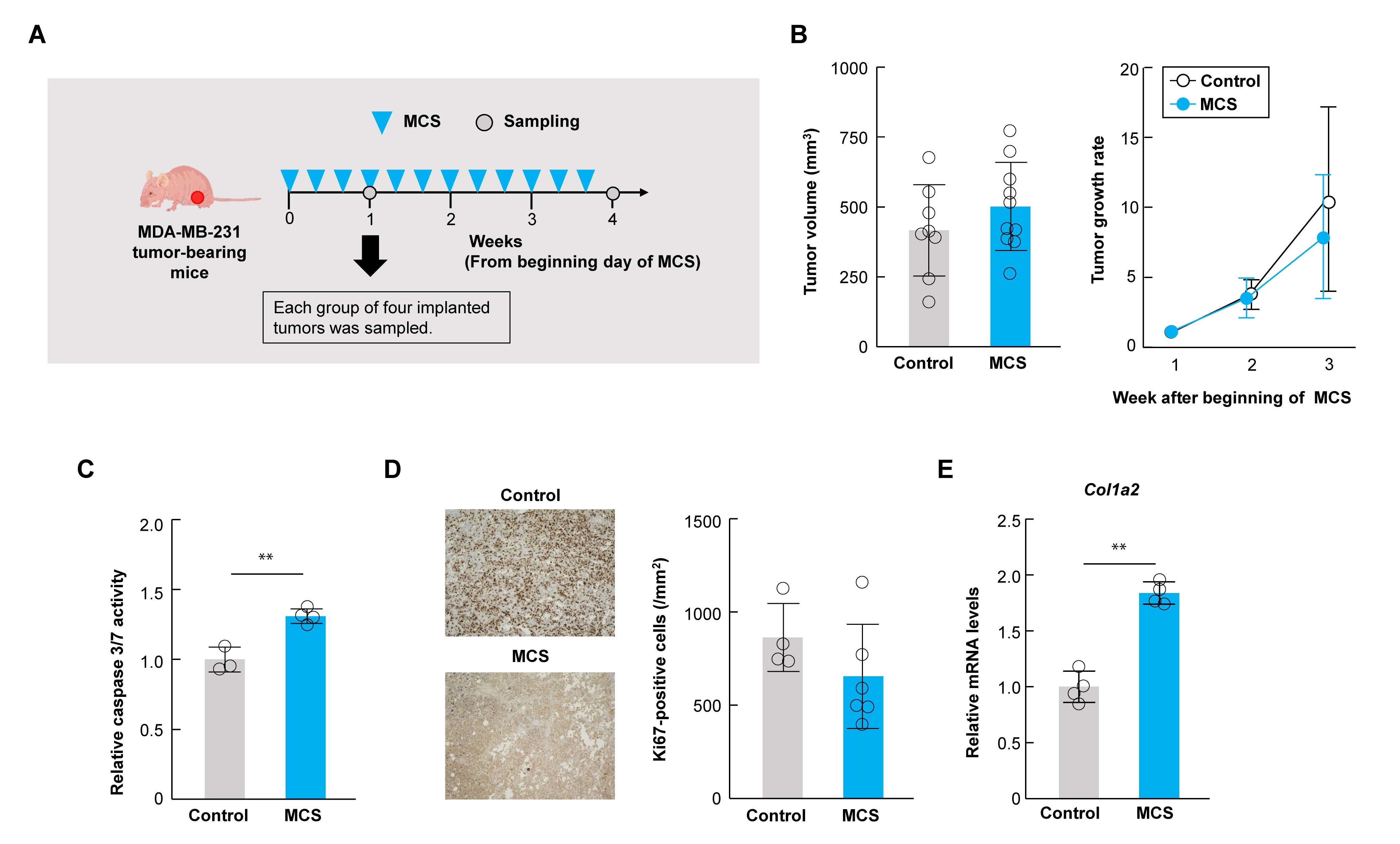


**Figure S11. Influence of MCS on MDA-MB-231 tumor growth in mice.**

**(A)** Schematic of the experimental procedure for MCS in MDA-MB-231-implanted mice. **(B)** Effect of MCS treatment on the tumor growth rate in tumor-bearing mice. MDA-MB-231 cells were subcutaneously inoculated into the fat pads of mice. The left panel shows the tumor volume for each group 1 week following the first day of MCS treatment. Values are presented as the mean ± S.D. (n = 9–10). The right panel shows the tumor growth rate for each group from 1 to 3 weeks after the first day of MCS treatment, with the tumor volume at 1 week after starting MCS treatment set at 1.0. Values are presented as the mean ± S.D. (n = 5–6). **(C)** Caspase-3/7 activity in non-MCS-treated and MCS-treated MDA-MB-231 tumors 1 week after the start of MCS treatment. Values are presented as the mean ± S.D. (n = 3–4), with the control group's value set at 1.0. ***P* < 0.01 indicates a significant difference between the two groups (*t_5_* = 5.843, Student’s t-test). **(D)** Immunohistochemical staining of Ki-67 in non-MCS-treated and MCS-treated MDA-MB-231 tumors 4 weeks after the first day of MCS treatment. Ki-67 staining was visualized using 3,3′-diaminobenzidine (brown), and nuclei were stained with hematoxylin (blue). Values are presented as the mean ± S.D. (n = 4–6). **(E)** mRNA levels of the fibroblast marker *Col1A2* in non-MCS-treated and MCS-treated MDA-MB-231 tumors 1 week after the first day of MCS treatment. The value for the non-MCS-treated group is set at 1.0. Values are presented as the mean ± S.D. (n = 4). ***P* < 0.01 indicates a significant difference between the two groups (*t_6_* = 9.705; Student’s t-test).


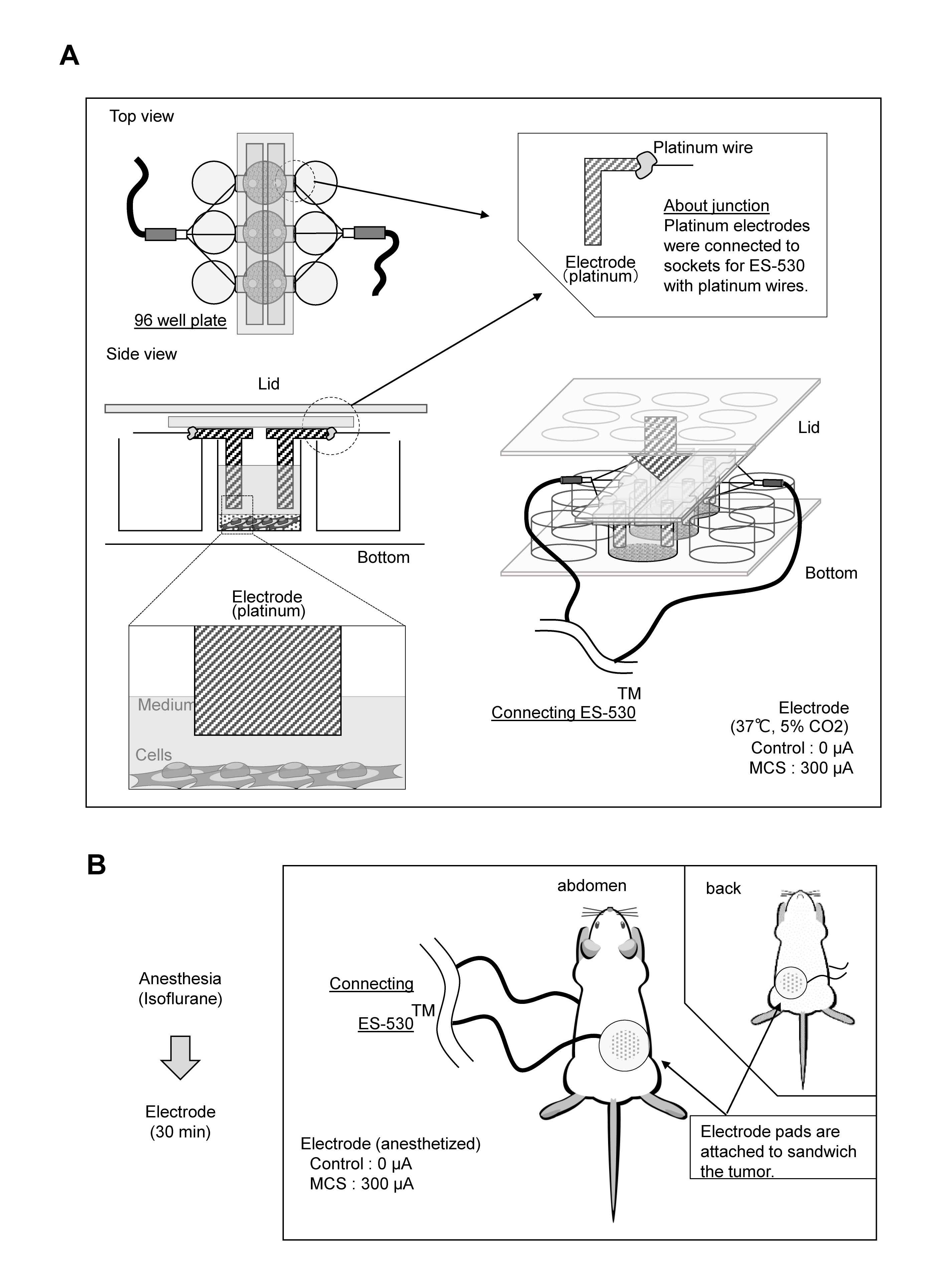


**Figure S12. Establishment of the MCS system for treating cells and mice.**

(A) Illustration of the *in vitro* MCS system components. The MCS system, utilizing ES-530^TM^ (Ito Co., Ltd., Tokyo, Japan), was used to treat cultured cells through platinum electrodes. (B) Illustration of the *in vivo* MCS system components. Mice were anesthetized and treated with MCS via sticky electrodes (Φ 32 mm × t 1.3 mm) connected to the ES-530^TM^ on their abdomens and backs.

**
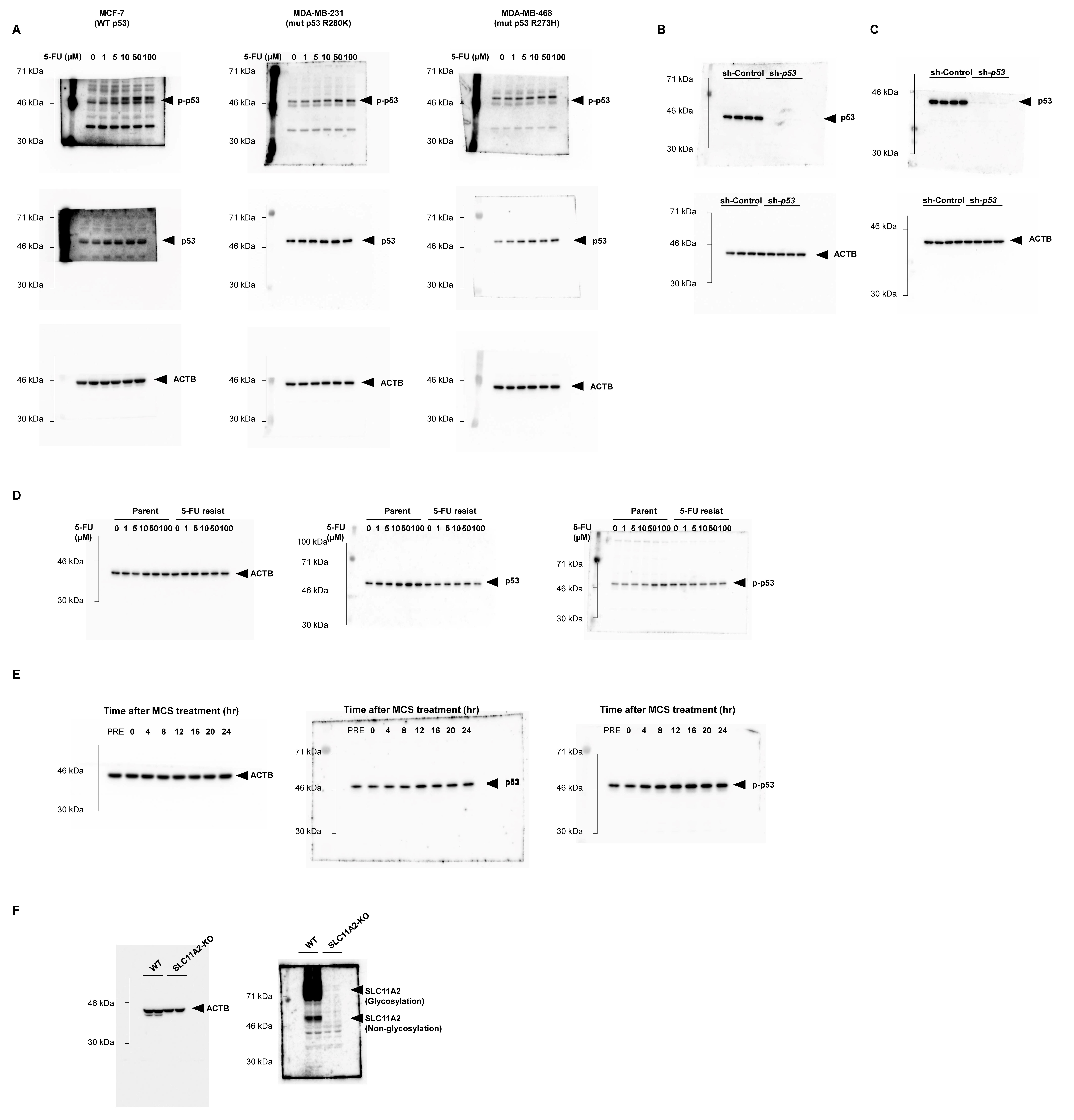
**

**Figure S13. Uncropped images of western blots.**

**(A)** Protein expression of p53 and phosphorylated p53 (p-p53) in MCF-7, MDA-MB-231, and MDA-MB-468 cells after treatment with 5-FU at concentrations ranging from 0 to 100 µM for 48 h, as shown in **Figure 1B**. **(B)** Protein levels of p53 and ACTB in MDA-MB-231 cells transduced with either sh-Control or sh-*p53* lentivirus, as shown in **Figure 2I**. **(C)** Protein expression of p53 and ACTB in MDA-MB-468 cells transduced with either sh-Control or sh-*p53* lentivirus, as shown in **Figure 3M**. **(D)** Protein expression of p53 and phosphorylated p53 (p-p53) in parental or 5-FU-resistant MDA-MB-231 cells after treatment with 5-FU at concentrations ranging from 0 to 100 µM for 48 h, as shown in **Figure 5B**. **(E)** Protein expression levels of p53 and phosphorylated p53 (p-p53) in MDA-MB-231 cells following MCS treatment, as shown in **Figure S5**. **(F)** Protein expression levels of SLC11A2 in SLC11A2 knockout MDA-MB-231 cells, as depicted in **Figure S8A**.

**Table S1 Primer sequences for human BAX promoter region**

| **Gene** | **Primer** |
| --- | --- |
| Human *BAX* promoter region (992 bp)  Forward  Reverse | 5′- CCGCTCGAGCCCATGTAAACACCATTCAGA -3′  5′- CATAAGCTTTCCCCGGACCCGTCCATCACC -3′ |
| Human *BAX* promoter region (204 bp)  Forward  Reverse | 5′- CCGCTCGAGGATTGGACGGACGGCTGTTGG -3′  5′- CATAAGCTTTCCCCGGACCCGTCCATCACC -3′ |

**Table S2 Primer sequences for quantitative PCR analysis.**

| **Gene** | **Primer** |
| --- | --- |
| Mouse *Col1a2*  Forward  Reverse | 5′- AAGGGTGCTACTGGACTCCC -3′  5′- TTGTTACCGGATTCTCCTTTGG -3′ |
| Mouse *Actb*  Forward  Reverse | 5′- ACTGTCGAGTCGCGTCC -3′  5′- CGCAGCGATATCGTCATCCAT -3′ |
| Human *BAX*  Forward  Reverse | 5′- CCCGAGAGGTCTTTTTCCGAG -3′  5′- CCAGCCCATGATGGTTCTGAT -3′ |
| Human *CD44*  Forward  Reverse | 5′- TGCAGGTATGGGTTCATAGAAG -3′  5′- GGTGGAGCTGAAGCATTGAAG -3′ |
| Human *NOXA*  Forward  Reverse | 5′- GGAGACAAACTGAACTTCCGGCA -3′  5′- GGCACCCATGAATGCACCTTCA -3′ |
| Human *PTGS2*  Forward  Reverse | 5′- CTGGCGCTCAGCCATACAG -3′  5′- CGCACTTATACTGGTCAAATCCC -3′ |
| Human *PUMA*  Forward  Reverse | 5′- GACCTCAACGCACAGTACGAG -3′  5′- AGGAGTCCCATGATGAGATTGT -3′ |
| Human *SLC11A2*  Forward  Reverse | 5′- ATCGGCTCAGACATGCAAGA-3′  5′- TTCCGCAAGCCATATTTGTC -3′ |
| Human *18s Ribosome*  Forward  Reverse | 5′- CGGCTACCACATCCAAGGAA -3′  5′- GCTGGAATTACCGCGGCT -3′ |

**Table S3 Primer sequences for ChIP analysis.**

| **Gene** | **Primer** |
| --- | --- |
| Human *BAX* gene p53 binding site  Forward  Reverse | 5′- GCATTAGAGCTGCGATTGGACG -3′  5′- TGAACGTGCGTCCTTCACGTG -3′ |
| Human *NOXA* gene p53 binding site  Forward  Reverse | 5′- CCTTCCCAACTCAAACACGATG -3′  5′- TCGAACTTGAAGAGTATGGGCAG -3′ |
| Human *PUMA* gene p53 binding site  Forward  Reverse | 5′- GAGGAGTTCCCAATGTTGCAAATGG -3′  5′- TCACCTTCCAGTGCCTAGTGTG -3′ |
